# Systemic Neurodegeneration and Brain Aging: Multi-Omics Disintegration, Proteostatic Collapse, and Network Failure Across the CNS

**DOI:** 10.3390/biomedicines13082025

**Published:** 2025-08-20

**Authors:** Victor Voicu, Corneliu Toader, Matei Șerban, Răzvan-Adrian Covache-Busuioc, Alexandru Vlad Ciurea

**Affiliations:** 1 Pharmacology, Toxicology and Clinical Psychopharmacology, “Carol Davila” University of Medicine and Pharmacy in Bucharest, 020021 Bucharest, Romania; victorvoicu@yahoo.com; 2 Medical Section, Romanian Academy, 010071 Bucharest, Romania; profciureaav@gmail.com; 3 Department of Neurosurgery, “Carol Davila” University of Medicine and Pharmacy, 050474 Bucharest, Romania; mateiserban@innbn.com (M.Ș.); razvancovache@innbn.com (R.-A.C.-B.); 4 Department of Vascular Neurosurgery, National Institute of Neurology and Neurovascular Diseases, 077160 Bucharest, Romania; 5 Puls Med Association, 051885 Bucharest, Romania; 6 Neurosurgery Department, Sanador Clinical Hospital, 010991 Bucharest, Romania

**Keywords:** neurodegeneration dynamics, signal transduction failure, proteostasis network collapse, TDP-43 proteinopathy, mTOR–autophagy axis, stress granule pathology, synaptic integrity breakdown, RNA-binding protein dysfunction, glial–neuronal signaling entropy, precision neurotherapeutic strategies

## Abstract

Neurodegeneration is increasingly recognized not as a linear trajectory of protein accumulation, but as a multidimensional collapse of biological organization—spanning intracellular signaling, transcriptional identity, proteostatic integrity, organelle communication, and network-level computation. This review intends to synthesize emerging frameworks that reposition neurodegenerative diseases (ND) as progressive breakdowns of interpretive cellular logic, rather than mere terminal consequences of protein aggregation or synaptic attrition. The discussion aims to provide a detailed mapping of how critical signaling pathways—including PI3K–AKT–mTOR, MAPK, Wnt/β-catenin, and integrated stress response cascades—undergo spatial and temporal disintegration. Special attention is directed toward the roles of RNA-binding proteins (e.g., TDP-43, FUS, ELAVL2), m6A epitranscriptomic modifiers (METTL3, YTHDF1, IGF2BP1), and non-canonical post-translational modifications (SUMOylation, crotonylation) in disrupting translation fidelity, proteostasis, and subcellular targeting. At the organelle level, the review seeks to highlight how the failure of ribosome-associated quality control (RQC), autophagosome–lysosome fusion machinery (STX17, SNAP29), and mitochondrial import/export systems (TIM/TOM complexes) generates cumulative stress and impairs neuronal triage. These dysfunctions are compounded by mitochondrial protease overload (LONP1, CLPP), UPR maladaptation, and phase-transitioned stress granules that sequester nucleocytoplasmic transport proteins and ribosomal subunits, especially in ALS and FTD contexts. Synaptic disassembly is treated not only as a downstream event, but as an early tipping point, driven by impaired PSD scaffolding, aberrant endosomal recycling (Rab5, Rab11), complement-mediated pruning (C1q/C3–CR3 axis), and excitatory–inhibitory imbalance linked to parvalbumin interneuron decay. Using insights from single-cell and spatial transcriptomics, the review illustrates how regional vulnerability to proteostatic and metabolic stress converges with signaling noise to produce entropic attractor collapse within core networks such as the DMN, SN, and FPCN. By framing neurodegeneration as an active loss of cellular and network “meaning-making”—a collapse of coordinated signal interpretation, triage prioritization, and adaptive response—the review aims to support a more integrative conceptual model. In this context, therapeutic direction may shift from damage containment toward restoring high-dimensional neuronal agency, via strategies that include the following elements: reprogrammable proteome-targeting agents (e.g., PROTACs), engineered autophagy adaptors, CRISPR-based BDNF enhancers, mitochondrial gatekeeping stabilizers, and glial-exosome neuroengineering. This synthesis intends to offer a translational scaffold for viewing neurodegeneration as not only a disorder of accumulation but as a systems-level failure of cellular reasoning—a perspective that may inform future efforts in resilience-based intervention and precision neurorestoration.

## 1. Molecular Collapse in Context: From Complexity to Catastrophe

Neurodegeneration occurs not as a sudden implosion of the cellular circuit, but as a gradual, ordered collapse of molecular coordination. For decades, research has documented the histopathological correlates of neurodegeneration: myloid plaques, neurofibrillary tangles, α-synuclein deposits, and the mislocalization of TDP-43 into the cytoplasm. While these aggregates have been identified and constitute a histopathological diagnosis, these aggregates are often not the most important source of the pathology inflicted [1]. The pathology may exist in the upstream processes—more specifically, the degradation of adaptive control systems that uphold intracellular organization, network communication and cellular self-identity over time [2]. The cascade of disintegration occurs on multiple levels of molecular complexity: signal transduction dysregulation, disturbances in gene expression control, failures in protein quality control, imbalances in metabolic homeostasis, and dependencies on feedback circuits between neighboring cells [3]. None of the levels of coherence are independent; they are functionally coupled and always co-evolving as adaptive regulation across many integrated levels of operation. As such, when a level of coherence is compromised, the effects of that disintegration can cascade across other levels, resulting in multiple, sometimes repeating rounds of destabilization and ultimately culminating in irreversible transitions of cell state, dissociation of circuits, and decline in cognitive or motor tasks [4].

At the early onset of discrete pathologies, maladaptive signaling dynamics are evident in neurons and glia long before neuronal loss becomes observable. In Alzheimer’s Disease (AD), phospho-proteomic studies of human hippocampal neurons have observed altered patterns of activation in signaling cascades of PI3K/AKT and MAPK. Persistence of ERK1/2 positive phosphorylation (Thr202/Tyr204) was observed alongside the suppressed phosphorylation of AMPK (Thr172) during stress, indicating a pathological metabolic interpretation of the stressor [5]. Similarly, in Parkinson’s Disease (PD), maladaptive AKT-GSK3β signaling can be observed in dopaminergic neurons, where persistent Y216 phosphorylation of GSK3β is combined with a loss of β-catenin translocation to the nucleus; this facilitates apoptotic favor and the destabilization of the Wnt-mediated transcriptional programme. These aberrations often occur in spatially constrained microdomains—postsynaptic densities, mitochondria-associated ER membranes (MAMs), or perinuclear nuclear laminae—where local environmental gradients (e.g., calcium flux, redox state, ATP availability) modulate the interpretation of the signaling event [6]. As proximal contextual parameters dissipate, these regulatory modules begin to exhibit pathological bifurcation: hyperactivation in certain circuits, and a resistance to signaling in others. This desynchronization can be understood as a loss of temporal fidelity at a larger scale—a failure in phase-coupled or circadian integration of signaling events that is necessary to support the synaptic plasticity and metabolic calibration that underlies healthy development [7].

In parallel with this signaling derailing, the specificity and structure of epigenetic regulation starts to fade away. Genome-wide sequencing of chromatin accessibility using ATAC-seq and ChIP-seq have illustrated parallel findings in AD and frontotemporal dementia (FTD): diminished chromatin accessibility at neuronal enhancers en-marked by H3K27ac and H3K4me1, and particularly at loci that direct synaptic vesicle cycling and neurotrophin response [8]. Recent research illustrates an age-dependent increment in variability in DNA methylation (methylation entropy) in REST and BDNF promoters; 5-hydroxymethylcytosine (5hmC) resulting from environmental demethylation from TET2 or TET1 is also diminished in neural stem cell niches and activated microglia [9,10]. In our view, changes in this respect signal not merely a toggle of gene expression but also a slowly growing erosion of transcriptional precision, resulting in the production of cell states that are noisy, unstable, and subject to maladaptive fate shifts.

In fact, one of the emergent principles across ND is the loss of state fidelity: that is, the inability of cells to maintain a clear molecular identity and lineage-specific functional programs. Using single-nucleus transcriptomic reconstruction of ALS and AD brain, neurons and astrocytes are highlight that exhibit transcriptomic signatures indicative of developmental, stress-response, and even activated glial paradigms--pointing to experience-dependent transcriptional convergence toward undifferentiated, mixed phenotypes [11,12]. Some microglia exist in mixed M1/M2 activation states, representing the co-expression of pro-inflammatory and phagocytic facets. The astrocytes of the AD entorhinal cortex up-regulate the expression of both A1 neurotoxic factors (e.g., C3, Serping1), as well as A2 reparative factors (e.g., S100a10, Tgm1), which signifies the astrocytes’ inability to resolve into stable functional modes [13]. The erosion of the epigenetic context will have an impact on proteostasis. Transcription of the expression of heat shock proteins (e.g., HSPA1A, DNAJB1) and proteins governing autophagy (e.g., BECN1, ATG9A) are lowered which directly results in decreased capacity to tolerate amounts of unrefolded proteins (misfolded) [14]. In the early AD cortex, mismatching proteomics–transcriptomics supports the idea that ribosomal biogenesis and the clearance of synthesized/translated protein inputs are uncoupled, producing translational overload and cessation of lysosomal degradation pathways. Meanwhile, mitochondria (metabolically stressed by PINK1-Parkin mediated mitophagy failure; calcium driven fission) languish and lyse around the perimeter of the nucleus, increasing ROS, which silently injures the integrity of the nuclear chromatin loop scaffolding [15]. The neuroimmune interface—specifically, the cross talk between microglia–astrocytes–endothelium—appears to be particularly important for inciting loss-of-function collapse. Inflammatory stressors (interleukin (IL)-1β, TNF-α, and damage-associated molecular patterns (DAMPs) from axons disrupted locally) induce the NLRP3 inflammasome activation; ASC speck formation and caspase-1 activation drive IL-18 release and pyroptotic cell death [16]. At least, equally importantly, the inflammasome activation drives histone acetylation (e.g., H3K27ac) that re-organizes chromatin in all glia, experiencing transcriptional reprogramming, which is related to NF-κB-dependent enhancer reshuffling. This drives self-reinforcing inflammatory signaling—a feed-forward lock-in loop—wherein signaling, transcription, and the chromatin state act to amplify each other toward rigidity and the loss of adaptable functional capacity. From an anatomical view of collapse, it is not uniform [17]. High-resolution spatial transcriptomics of the entorhinal cortex, substantia nigra and hippocampus marry regional patterns of degenerative vulnerability to gradients of mitochondrial density, chromatin accessibility, glial heterogeneity and vascular proximity. In the aged hippocampus, the impaired AQP4 polarization in perivascular astrocytic endfeet irreversibly interfere with glymphatic clearance and subsequently promote the accumulation of tau, α-synuclein and RNA granules, escalating normal levels of metabolic stress and increasing cytoplasmic crowding [18]. Collapse, then, has topological propensities and is shaped by the local, structural context and by the history of sequences of exposure to inflammatory or metabolic disruption [19].

An extensive body of work supports re-conceptualizing neurodegeneration as an ordered failure of biological control—a molecular collapse architecture in which multiple adaptive systems have exceeded their compensation limits and destabilized into dysfunctional attractor states. Each system that regulates processes (ssignaling, epigenetics, proteostasis, immunity, and cell fate) is not only impaired but destabilized through intersystem feedback, energy shortfall, and transcriptional noise accumulation. In addition, the effects are far less static: they progress, they spread, and they will be more resistant to endogenous repair unless redirected through precise therapeutic intervention [20].

This review aims to track the invisible architecture of neurodegenerative collapse—an architecture often seen in fragments but not often assembled into a coherent framework. Rather than providing a catalogue of pathological characteristics, the narrative seeks to represent the internal logic of collapse: how specially calibrated molecular signals begin to smear, the ways in which networks suffer de-coherence without identified damage, and the slow erosion of resilience that is only apparent once symptoms emerge. The following sections elaborate this process through interweaving layers of regulatory failure. These include disorganized intra-cellular communications, epigenetic drift, maladaptive immune signaling, impaired clearance systems and the continued demise of cellular identity fidelity. Some key points will be emphasized—specifically, intersecting sites of multiple stressors, and points of inflection at which a biological system is at the transformational edge of adaptation or collapse.

In bringing these domains into conversation, this review seeks to neither reduce complexity, nor to expunge ambiguity, but rather to bring complexity into a corporeal view and to provide the reader with a map through which similar mechanisms might be reconceptualized, or unobserved registers of coherence might be amplified. Taken together, the map will facilitate reflection, foster new hypotheses for study, and enhance perceptions of the loss of coherence in degenerating brains and, potentially, recovery.

## 2. Disintegration of Signaling Networks in Neurodegeneration

Signal transduction in the nervous system does not only encode molecular affector responses but constructs the functional interpretation of exceedingly complex biochemical and environmental states. The fidelity of the signal transduction in molecular processes depends on spatially and temporally traditional precision, scaffold integrity, receptor compartmentalization, isoform diversity, and adaptive feedback regulation [21]. In ND, this critical interpretative understructure decays in a progressive manner. What initiates as slight changes in the amplitude or duration of the signals evolves into gross desynchronization, spatial misrouting, feedback decoupling, and finally a loss of signal logic. Ultimately, measurements of ND gait may resemble the slow uncoupling of complex processes associated within pathological phases in defunct signal logic [22].

In the preliminary phases of ND transitions, neurons exhibit an unstable signal decode fidelity; however, this occurs relatively early in the initiation of the consequential biological programs within trophic, metabolic and stress-response signaling systems [23]. In AD, hippocampal pyramidal neurons show decreased IRS-1 stem tyrosine phosphorylation while showing also a higher level of serine phosphorylation regulation (i.e., ser312) mediated through stress-kinases e.g., JNK and kinases IKKβ, post-translationally modifying a ligand. These shifts abolish PI3K activation and translate into a decrease in Thr308/Ser473 AKT phosphorylation [24]. Confounding downstream events decrease or completely eliminate the ability of the pathway to properly inhibit GSK-3β via S9 phosphorylation, leading to the hyper-phosphorylation of tau at normalized epitope sites: S396, T231, S262. This leads to the disruption of micro-tubule stability and contains pre-tangles. At the same time, mTORC1 becomes chronically hyperactive due to Ragulator-Rag GTPase disassembly, which causes the indirect oxidative inactivation of the tuberous sclerosis protein (TSC) complex [25]. This predisposes the pathway with hyperactive S6K1 signaling without any associated increase in autophagic flux, thus creating an untenable translational load on neurons; this leads to ER stress, a saturating UPR, and ongoing proteostatic lack of maintenance [26].

A corresponding attenuated frame of collapse is constructed in PD by dopaminergic neuron chronic PLC to cause calcium entry via L type Cav1.3 channels. This overload also activates calcineurin and drives pro-survival transcription factors, such as MEF2D, to dephosphorylate and translocate NURR1 to the nucleus, all of which are required for mitochondrial biogenesis and antioxidant defenses [27]. Furthermore, DJ-1 oxidation prevents the inhibition of PTEN to decrease AKT activity, while hyperactive GSK-3β hastens the onset of increased FOXO3a concentrations in the nucleus and pro-apoptotic gene expression, exacerbating the vulnerability of the neurons. However, in these pathways, which are altered in both diseases, the effect of isolated actions of the pathways is somewhat irrelevant because of the ways in which these pathways are dysfunctional, as well as the changes to the intracellular logic of how the neurons interpret trophic support, the energetic state, and stressors in their environments [28,29,30]. Subcellular disorganization adds to this misinterpretation. In dendritic spines, Aβ oligomers enhance the calpain-mediated cleavage of PSD-95, the main scaffold for anchoring NMDA receptors and CaMKII, which abolishes the local signaling hubs required for synaptic potentiation and CREB phosphorylation, functionally disabling activity-dependent plasticity at the dendritic spine [31]. In the axon initial segment (AIS), the loss of Ankyrin-G and clustering of Nav1.6 due to TDP-43 pathology negatively impacts action potential initiation reliability and axonal excitability. MAMs that are structurally dissociated by mutant presenilin-1 and tau pathology remove components of IP3R3–GRP75–VDAC1 and detract from ER–mitochondrial calcium transfer; at the same time, they drastically decrease the ATP production capabilities and buffering action of the two organelles [32]. If dysregulation of mTORC1 is not a sufficient obstacle, its effect is further disentangled by preventing the nuclear translocation of TFEB and lysosomal biogenesis, and halting autophagic renewal altogether [33].

At the same time, the derailment of lipid-mediated signaling contributes to ongoing cellular disorganization in terms of the interpretation of cues in their environment. The disruption of sphingolipid metabolism, most notably the accumulation of sphingolipids-derivates and especially ceramide derived from hyper-active sphingomyelinases, inhibits PKCζ, the main bin of polarity and neuronal survival; this is in addition to their focal role in outer mitochondrial membrane permeabilization [34,35]. Lipid raft microdomain structural integrity is compromised, resulting not only in the dismantled compartmentalization of their downstream signals in receptor systems such as TrkB (Trk tyrosine kinase receptor 2) and TREM2 (triggering receptor expressed on myeloid cells 2) but also in the absence of caveolin-1 in the glial membrane, which specifically influences the dynamics of their calcium-coupled signaling and regulation of exosomes [36]. Together, these faults are more than just an abnormality of intracellular signaling; they dismantle neuron–glial communication and instigate a chronic paracrine layer of mala-adaptation feedback. Moreover, all of the structural differences are exacerbated by all forms of temporal desynchrony. While signaling pathways are periodic, the physiological circadian or periodicity of the signaling pathways degrade as neurodegeneration progresses [37]. In the AD cortex, SIRT1 (silent-mating-type information regulation 2 homolog 1) deacetylation of BMAL1 (brain and muscle ARNT-like protein 1) declines dramatically and NAD+ (nicotinamide adenine dinucleotide) synthesis also declines, resulting in more muted oscillations of PER2 (period 2) as AKT (serine/threonine-protein kinase) and AMPK (AMP-activated protein kinase) circadian rhythms collapse; within the hippocampus, the temporal variation in CREB (cyclic AMP Response Element-Binding) activation disappears under diurnal conditions that decrease the ability to consolidate memory [38]. Meanwhile, glial cells and astrocytes also lose, to varying degrees, the rhythmic expression of core clock genes, which causes them to lose their regular cycling of the glutamate transporters (e.g., GLT-1) and ultimately the disconnection of the activity characterized by the glymphatic clearance mechanism with the structure of sleep architecture. This degeneration of the metabolism-processing mechanism involved in the distinctly different phases of the circadian cycle describes the entrapment of a spiderweb of misplaced signaling, which then degrades the restorative actions of a night-mode function and synaptic scaling [39,40].

Single-cell multi-omics now affords the recognition of the individually heterogeneous progression of these failures for cell type and disease stage. Integration of scRNA-seq and phospho-proteomics for human postmortem cortical tissue provides clear evidence of subtype-specific signaling decay: For example, deep-layer excitatory neurons of the cortex have a much stronger ability to signal via MAPK pathways, but still less than expected given their transcripts for those receptors [41]. Microglial cells show greater reliance on their signaling senescence, with their increased burden of inflammatory signaling meaning that they rarely up-regulate their SYK or TREM2-DAP12 pathways. Their astrocyte counterparts report dissociation in terms of their Ca^2+^-NFAT and JAK-STAT pathways but, as expected, they also undergo shifts in terms of transcriptional noise and expected chromatin accessibility entropy [42]. The findings suggest that signaling breakdown is both stochastic and spatially deterministic, in that declines in signals generally occur at different rates across populations when subjected to common signals. These spatiotemporal failures will yield increasingly incoherent crosstalk across signaling pathways [43]. For instance, in the case of ALS, the DUSP and MKP mRNAs, which have E3 ubiquitin ligase properties, whose cytoplasmic inclusions TDP-43 forms would stop the termination of MAPK cascades, thereby maintaining sustained p38 MAPK phosphorylation, prolonging stress granule duration, and accelerating nuclear lamina disassembly and integrated stress response activation [44]. Failing dynamical changes in nuclear import pathways, it would also block the nuclear retention of phospho-STAT3 and phospho-CREB in nonspecific and/or ectopic transcriptional scenarios where transcription is either inactive or chronically active [45]. In the case of FTD, IL-1β stimulation turns on MyD88–NF-κB pathways while inhibiting the transcription of IL-10 and BDNF at the chromatin through HDAC2 and REST, thereby blocking the anti-inflammatory IL-10 and neurotrophic BDNF response [46]. Wnt signaling would also be inhibited by inducing DKK1, with upregulation driving decoy receptor signaling formation through SFRP1 and not allowing β-catenin accumulation, despite the persistent expression of associated Wnt ligands. Defect activation of the attenuation process will further amplify dysfunction [47]. Signal amplification through the ROS-mediated oxidation of specific cysteines in PKC catalytic domains would induce limitations, reactivating phosphatases such as PP2A and Calcineurin while encroaching activation from JNK and ERK, which activate CDK5–p25 and hyper-activate phospho-Tau phosphorylation and histone H1 modification or chromatic compaction; this is well known to have inhibitory effects on transcriptional elongation through the ser2 phosphorylation of Pol II CTD [48,49]. For example, phospho-CREB, failure to bias transcription after transient nuclear accumulation, and failure to recruit a CBP after phorphorylation due predominantly to HAT inactivity, when hypoacetylated, fail to leave in part and leads to a failure of signaling ending. Simultaneously, a degradation that limits a handful of specific PKC isoforms (i.e., PKCε, PKCδ) demands processes of signaling modulation directed at DAG- and calcium-dependent forms of signaling that are limited to synaptic saturating states [50]. Problems with endosomal and exosomal signaling could continue to amplify incoherence. For example, hyperactivation of Rab5 induces early endosomal enlargement that initiates delays in TrkB receptor and TREM2 recycling to clinically relevant antigen-antibody complexes, blocking dissociated re-engaged BDNf and its anti-inflammatory ligand [51]. Even less conducive would be the Rab11 dynamics that would not permit element recycling to synaptic membranes, thereby diminishing the chance of producing LTP-associated potentiation by only weakly associating with TrkB [52]. In this light, exosomes secreted in astrocytes from amyloid-β (Aβ) mixed AD models, which are virtually distinct in expression and whose differential profiles of miRNAs and protein cargo will be more strongly characterized for the pyrrhic loss of a neuroprotective miR-132—around two-fold stronger. Moreover, the gain of a pro-inflammatory miR-155 propagates an inflammatory bias to the receiving neuron by propagating a noxious environment.

Additionally, the degradation of endolysosomal compartments within acid appreciation will also repro-model aberrant conditions involved in receptor degradation and truncated inhibitory durations of cellular signaling and the capsized chaos of reverse feedback. Aging neurons become increasingly less neurally and spatially distinct because of the loss of functional AKAP assemblies’ co-operative association with L-type calcium channels, which offers less capacity to find bias towards PKA specificity, or systematically distinct in terms of synaptic activity modulation [53]. In this reduction, once the signal does not terminate, modulate, or have spatial specificity, there is a chance for bio-cellular entropy. The stability of the system can fail from the neurobiological system periphery-of-silence, which is deeper if there is an increasing amount of nominally possible coherent noise, e.g., persistently ongoing signals in this context appear contextually irrelevant, neurasthenic in spatial orbit, and with no helpful outcome [54]. For example, at a fastened systems participation level, chaos collapse may be read as a loss of information entropy: typical neurons represent eld or codiscriminate coherence. Otherwise, loud signals can appear through the compression of input to produce coherent molecular responses, or specified outputs that are never sacrificed; this is a consequence of a stimulus of maximal discrimination efficiency and signal to noise implicature, fully in space and time [55]. The wandering transitions of normal/early numbing increase neurodegenerative signaling confusion by being commonly characterized at higher basal activity, or an atypically capable state of degeneration is seen by not stopping common inhibition and trampling cognitive practice or modality [56].

All of this erodes discrimination, but the system also tacitly forfeits the opportunity to conditionally respond and is very fast to weaken shared information about the distinction in a network of dynamic stimulus-response connections. As a result, once again, neurodegeneration represents itself incorrectly separately, as systems of regulation but also as interdependent liability states, whether systemic or unstable or otherwise chronic liability situations work to create a formerly active hallmark of the bedrock. Thus, chaotic processes continue to develop into self-regulating features of information motion where neurons can work themselves in decreasing collective autonomy; as such, when we say ‘neurodegeneration,’ we must recognize and accept that we are demonstrating or exhibiting proverbial damage but demonstrating interpretive failure. This represents increased difficulty, forwarding action for cellular processes and functions in order to create homeostasis in cellular processes and functions in a comparatively healthy space, and cells in distressed canine spaces. To better systematize the critical points in signaling collapse, Table 1 summarizes those salient molecular nodes whose dysfunction becomes convergence points in neurodegenerative conditions. These “decision-point nodes” assimilate different upstream stressors, cell-type specific vulnerabilities and modulatory layers, which organize their pathological course. We hope to also broadly map reversibility potential and current intervention strategies, which may serve as a conceptual scaffold for addressing signaling breakdowns with the potential for issue-specific denotation.

## 3. Epigenetic Drift and Transcriptional Entropy in Neurodegeneration

Epigenetic regulation, once achieved in a mature central nervous system (CNS), is a multilayered epigenetic interface that organizes experience, metabolism, synapses, and long-term cellular identity. The real-time epigenetic architectures differ from transitory signaling cascades in that they impose engagement threshold regimes that transpose input histories while having the capacity to modulate aspects of the genome over time and space [87]. The grammar of epigenetic architectures, via a range of chemical signals and structural scaffolds (e.g., methylated cytosines, histone tails and their diverse range of functionalities, 3D chromatin looping, and a whole ecosystem of non-coding RNA), supports stabilized fates of cellular population identities and plasticity and models transcriptional engagement within physiologically context-driven parameters [88]. With the logic of neurodegeneration, an epigenetic interpretive logic cannot simply be weakened: rather, it has been coupled. A new emergent state has begun in which the entire set of inputs has lost any contextual relevance to each other, thresholds seem to misalign, and regulatory feedback has become chaotic. Some of these phenomena are referred to as transcriptional entropy, a condition characterized by an emergent degree of degenerative change in the information theoretic fidelity of the machinery of the genome to engage the most relevant responses of the genome.

Important genomic datasets from genome-wide methylation profiling suggest not only that there are some local epigenetic implications, but that this state is one of emergent pan-topological prescription [89,90,91]. In the case of AD, regarding the decay of long-range intergenic contacts (especially those expressed and canonical contacts as part of the CTCF-architectural bound loops in the neurons of entorhinal cortex),there are also issues of enhancer–promoter de-coupling and ectopic enhancer activation [92,93]. The dissolution of chromatin loops appears to be disconnected from amyloid canonical pathophysiology and instead correlates to a range of age-based dissociative impacts on cohesin stabilization, oxidative stress, and pacing shifts in nucleosomes [94]. In addition, regions of high contact entropy also have an abundance of transcript noise in single-nuclei RNAseq datasets—one may suggest a mechanistic correlational fidelity between 3D genome topology and regulatory coherence [95]. The disintegration of transcription factor (TF) binding logic would exacerbate an escalation of entropy. In instances of neurodegeneration, cis-regulatory grammar—the spatial and combinatorial arrangement in TF binding sites—becomes disorganized [96]. For example, in neuronal super-enhancers, there is depletion of TF binding site density and clustering for CREB, MEF2, and SRF, and, as a result the transcriptional circuits’ capacity to buffer, the consequences of signaling are disrupted. The TFs in these enhancers fail to integrate inputs, misalign the time of gene activation, and fail to become activated even with upstream stimulation. At the same time, chromatin-modifying enzymes such as KMT2A (MLL1), BRD4, and EP300 are mis-routed in different tauopathy models to either eu-nuclear or nucleolar compartments, which fuel enhanced TWNI productivity into stimulus-responsive enhancers [97]. In replicating gene expression, the very integral aspect of the timing associated with gene expression is decoupled from the stimulus; this cohort of genes would normally follow a temporal schedule incited by the stimuli. As if that were not exacerbated by this dysregulation, KMT2A (MLL1), BRD4 and EP300 were routed as bivalent domain, meaning there was persistent co-enrichment of H3K27me3 and H3K4me3 at neurodevelopmental loci, suggesting, for example, that neurons sitting in excessive levels of co-enriched H3K27me3/H3K4me3 have mismatched (plasticity) programs which could be aberrantly dealt with (reconstituted) later in neurodevelopment under heterogeneous conditions, and regardless of the downstream context [98]. Thus, these commensalities of developmental plasticity from the epigenetic memory banks of early neurodevelopment, relatedly, produce the potential for misexpression [99].

Neurodegeneration deals a similar blow to the synchrony of epigenetic synchrony that pertains to different states existing as distinct types. Agents can exist in “stressful states” asynchronously, as distinct lineages such as astrocytes, oligodendrocyte precursor cells and microglia, and as mutually disruptive temporal peers, disrupting transcriptional coordination at the tissue level with other cell types and, as such, disrupting transfusion-level transcriptional coordination in response to completeness as a tissue-level tissue complex. Next, glial sub-populations began to adopt transcriptional drifts toward hybrids—partially inflammatory, but partially reparative—and are incapable of the completion of either coherent trajectory. At this point of network-level incoherence—either contours captured in aggregate gene expression or measurements of the transcriptomic drift of sub-populations—there should now be measures of increasing inter-cellular variance and entropy [100,101].

**Figure 1:**
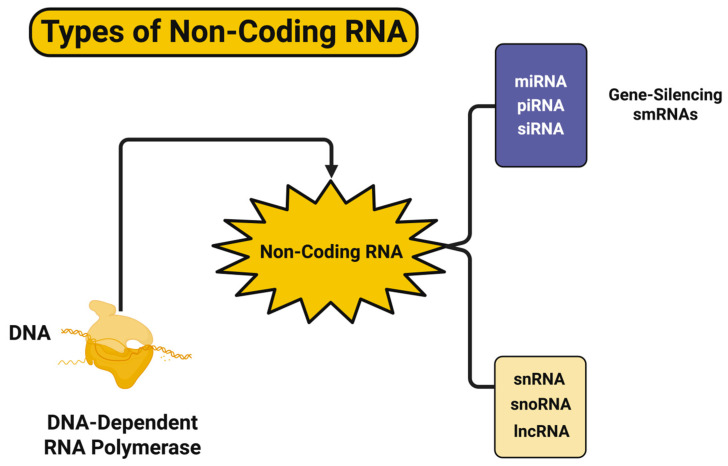
Illustrative classification of non-coding RNAs. Gene-silencing small RNAs (miRNA, piRNA, siRNA) and structural regulatory RNAs (snRNA, snoRNA, lncRNA) derive from transcriptional activity and contribute to diverse functions, including chromatin remodeling, splicing, and mRNA regulation. These categories are variably affected in the context of neurodegenerative disease, where epitranscriptomic dysregulation appears to play a growing role.

Finally, the very logic of the nuclear space will begin to diminish. Epigenomic studies have provided evidence that the dissociation of the lamina-associated domain (LAD) and the integrity of the topologically associated domain (TAD) allows abnormal enhancer–promoter interactions to occur. Nucleocytoplasmic transport system dysfunction delays the recruitment of epigenetic regulators [102,103]. In amyotrophic lateral sclerosis (ALS) and FTD, the misassemblage of nucleoporins, including NUP98 and NUP153, is observed. The misassemblage of nucleoporins can confine or allow the escape of chromatin-remodeling proteins, transcription factors, and small RNAs [104]. Recent evidence of ‘condensate failure’ involves the disruption of phase-separated nuclear bodies, such as paraspeckles and speckles, through the loss of individual modular identities. The assumption is that ‘condensate failure’ would limit the compartmentalization of regulated bursts of transcription [105]. Additionally, epitranscriptomic instability at the RNA level support chaos. For example, unregulated METTL3 localization and the misdistribution of the reader proteins (e.g., IGF2BP1, YTHDF1) generate m6A methylation that designates transcript lifetime and translational response [106]. Similarly, RNA-binding proteins (RBPs) (e.g., FUS, TDP-43, ELAVL2) also lose fidelity, including their propensity toward aggregates, which hinder key transcripts. The loss of the releasability of transcripts generates random inclusion or exclusion variability in alternative exons for key synaptic and metabolic genes. For example, the majority of observable RBP dysfunction in ALS samples amassed within the cerebellum and motor cortex, and it was apparent early in disease progression, before gliosis and neuronal loss; this suggests that RBP dysfunction is an early pathological indicator. Recent single cell transcriptomic and spatial transcriptomic analyses demonstrated a prominent feature: attractor state erosion [107]. In order to contextualize this kind of dysfunction, it is useful to consider the larger framework of non-coding RNAs (ncRNAs) that modulate epigenetic and transcriptional regulation. This includes small RNAs that transcriptionally silence gene expression (e.g., piRNA, miRNA, siRNA) and longer non-coding transcripts (e.g., lncRNA, snRNA, snoRNA) that can influence chromatin structure, splicing and the nuclear architecture, etc. [108]. An increasing number of studies investigate further dysregulation in these families in the context of ND. The diagram in Figure 1 illustrates the types and families of ncRNAs, as well as their origins and broader functions within the RNA regulatory continuum—and many of these are dysregulated in disease.

Generally speaking, cell types take up distinct attractor space, and transcriptomic identities still have a predictable level of variability. In neurodegeneration, the wells of attractor states are flattening. The cell populations do not return to steady gene expression after perturbation; rather, they drift randomly in the gene expression space. Shannon Entropy and mutual information analyses can offer evidence of greater stochasticity and decreased regulatory constraint [109].

This drift also occurs in time, i.e., genes that used to be regulated by very specific perturbations with specific timing now have delayed, dampened, or asynchronous activation. This desynchronization allows the stress response to separate from biological intention, especially in synaptic scaling, oxidative stress detoxification, and immune checkpoints. Having a disrupted genomic response may incur cumulative errors, despite the signaling systems being intact [110].

As a consequence, therapies will not just need to go beyond reactivating or silencing the gene targets. The reactivity of the genome may need to become a coherent genome-adjustment, i.e., logical connections between environmental signals, chromatin accessibility, TF engagement, transcript processing, and translational activity [111]. These approaches may include the CRISPR/Cas9-based reprogramming of enhancer logic, RNA-directed therapeutics that may restore RBPs engagement with transcripts, and chronic renewal of phase-separated parts of the nucleus that segregate transcription. It may require repeated systemic renewal-in-extended-space of the chromatin, RNA, and spatial compartmentalization of the nucleoplasm to re-enable the dynamic responsiveness of the genome to perturbations in the environment [112,113]. To sum up, an increase in transcriptional entropy and the disintegration of temporal coherence equates to a disruption of regulatory processes, or of the rationality/logic that integrated the signals/indicators into coherent biological activities within the genome.

## 4. Proteostatic Collapse and Organelle Overload in Neurodegeneration

Proteomic integrity is vital to neuronal survival, synaptic integrity, and adaptive plasticity. Neurons have distinct characteristics compared to actively dividing cells, as neurons maintain long-lived proteins and organelles over decade-long timelines, and the standards by which proteostasis is satisfied are high. Broadly defined, proteostasis is the well-coordinated interdependent action of molecular chaperones, the ubiquitin–proteasome system (UPS), autophagic-lysosomal flux, spatially buffered degradation centers, and compartmentalized sites of quality control. It goes beyond a simple linear processing pipeline and reflects a broad spectrum of signaling dynamism [114]. The progressive dysfunction of the proteostasis network in instances of mid-stage ND is marked by a full occupancy proteome, saturation of important kinetic space, and deterioration of substrate sorting fidelity [115]. Some have suggested that proteostasis should be reconceptualized as a high-dimensional information-processing network rather than a distribution of linear degradation pathways. In this high-dimensional paradigm, cellular stress has generated enough inductive decision complexity that it has become simply a cost of cognition to rationally determine what proteins need to be folded, sequestered, and destroyed [116]. When a threshold of cognitive decision making is breached, the proteostasis system enters into a state of information entropy where the processes of substrate recognition become non-specific, degradation response times increase, and confirmatory activity becomes decoupled [117]. The degradation of the proteome from selective to unselective has recently been modeled in information theory (e.g., Shannon entropy, Kullback–Leibler divergence, signal estimation). These models support the hypotheses of “entropy biomarkers,” reflecting observable shifts in proteomic distributions, signaling early system drift towards a state of collapse [118].

The trajectory towards collapse of the proteome is unevenly spatially distributed. Spatial patterning of quantitative proteomic degradation is transcriptome biased. In the AD model system, the entorhinal cortex and locus coeruleus appear to be some of the first brain-knees to experience oxidative fragmentation of synaptic proteins, first seen in the failure to be protected by intact 20S proteasomal activity [119]. Either way, this means it is a failure of tagging and routing substrates through the smaller task of choice, not failure through clearance. Both iPSC-derived neurons and cerebral organoids currently indicate that specific subtypes of neurons do show differential susceptibilities in proteostatic resilience. Glutamatergic neurons have sensitivities to UPS inhibition and ER stress that others do not; therefore, they must have had adaptive mechanisms for early translation repression and other aggregate formation and processes, rather than for GABAergic or dopaminergic neurons [120]. Although these processes are dependent and critically reliant upon the ubiquitin topology and signaling from the inning, impaired E3 ligases losing target specificity or mislocalized E3 ligases may be interventional with their interactions with UBE3A, ITCH and other pathways. Mislocalized or improperly chain-length-managed deubiquitinases making ambgous modifications, such as USP14 or OTULIN, can totally stop all proteasomal process [121]. When these coalesce with the resistance time of stalled ribosomes and recycled failed ribosomal subunits, it can lead to the total failure of the ribosome-associated quality control (RQC) pathway [122]. Michal dead zones formed with the count of saturating translation unique mRNA transcripts, usually with G quadruplex motifs or repeat expansions, leading to their collisions with the other stalled ribosomes and exploding all triaging that is transitioned to them, overwhelming them with fractured polypeptides [123].

Macroautophagy is a a very efficient even compensatory system that is not missing viable alternation at the autolysomal fusion synthetic event. This is where we witness misassembled VAMP8, STX17, SNAP29 as the unrecoverable auto-lysosomal fusion competency failure; there is partial inhibited lysosomal acidification due to reverting the proton pump to its previous sub-cellular localization, as well as impaired lipid composition and the remaining maturation of cathepsins being arrested [124]. Additionally, chaperone-mediated autophagy (CMA) is unable to enter cytosolic α-synuclein oligomers to enlist onto the LAMP2A channels. There are many layers of these failures, ultimately (indirectly) becoming more autophagic, while technology is electively a globally ineffectual degradation responsible for both recycling and clearing soluble and insoluble aggregate structures [125]. Membrane contact site (MCS) ER and mitochondria or lysosomes are coordinate sites for stress signaling, lipid shuttling coordination, and calcium fluxes. The MCS disrupts the levels of scaffold proteins VAPB, MFN2 and ORP5, similar to the scaffolding process, eliminating synchrony in proteostasis signaling. There is also dysregulated MCS, increasing the noncoordination of processes that are all intricate factors, as in mitochondrial fission–fusion imbalance, induced increased ROS release during perinuclear assembly, and granules with accumulated abnormal redox signaling [126]. After prolonged/unfolded protein response (UPR) activation in the endoplasmic reticulum (ER), the pendulum swings back. The process shifts from adaptive to maladaptive construction. By inhibiting translational initiation, the subsequent activator transcriptional factor (ATF) 4 (the PERK-eIF2α-ATF4 cascade) provides transient eukaryotic initiation factor 2α (eIF2α) phosphorylation to relieve the burden [127]. However, a chronic stress scenario will ultimately bind an extended block in translation and move toward CHOP-related apoptosis instead. Significantly, single-cell transcriptomics from tauopathy models show cortical neuronal subsets, revealing paralogous ER stress sensors that are both engaged and active. Meanwhile, downstream integrated stress response (ISR) deployment is completely absent, indicating a decoupling of the stress sensor and the downstream response [128]. Mitochondrial proteostasis decouples as well. The translocase of the inner mitochondrial membrane (TIM)/translocase of the outer mitochondrial membrane (TOM) complexes have a tendency to destabilize and can shift the import of nuclear encoded respiration and quality-control-related enzymes. The mitochondrial proteases (CLPP and LONP1) simply cannot cope with the accumulating stressor. Stalled PINK1 stabilization, coupled with ineffective phospho-ubiquitin recovery with no lysosomal docking, makes mitophagy pointless [129]. These damaged mitochondria can also release immune-stimulatory DAMPs (for example, cardiolipin, oxidized mtDNA and ROS), which initiate astroglial and microglial inflammasome cascades [130].

Stress granules also lose their transient identity, although they continue to serve their original purpose of protecting stalled translation complexes. At present, phase transitions to irreversible aggregates. In the C9ORF72-linked ALS and FTD, pathologic stress granules nucleate from arginine-rich dipeptide repeats (DPRs) and sequester eIFs, importins, and nucleoporins (for example, Nup62) to stop the formation of both translation and nuclear transport. These irreversible aggregates are resistant to extraction, which inhibit chaperoning action and provide another local accumulation point in a streaking proteostatic overflow [131,132].

Through this process, there is also a full volley of several post-translational modifications (PTMs) that displace any substrate taking up the load. In addition to entering into the canonical PTMs, manipulations of constructs in noncanonical PTMs (for example., SUMOylation, crotonylation, and glutarylation) can all inhibit proteasome docking and chaperoning activity. These changes are implicated in the acetylation and metal processes, altering histones that change chromatin composition, creating nuclear stress as a result of the proteomic load, which leads to transcriptional collapse [133]. The cross-talk and compartment sharing of organelles is further impeded. Perhaps as a result of Golgi fragmentation from the inactivation of GRASP55 or BICD2 function, individual organelles (protein trafficking and lysosomal enzyme maturation) are inhibited [134]. Peroxisomes lose lipid processing and redox buffering functions in a down-regulation of membrane-bound receptors PE13 and ABCD1. Destroyed lysosomes leak iron and, through reprocessing, become redox-active and inflict ROS damage on neighboring compartments. One spatial proteomic mapping especially revealed the degeneracy of organelle borders to make vesicular mosaics, thereby obliterating compartmentalization [135]. To complement the detailed breakdown above, Table 2 outlines the key regulatory axes implicated in the collapse of proteostatic and organellar networks across neurodegenerative states. These axes represent converging points of genetic vulnerability, upstream stress signaling, and therapeutic reversibility potential.

In neurons, the web of proteomic identity control essentially disappears at the network level, where the referenced encoded logic determining what to keep or dispose of is frenetic in nature. The conditionality of an individual translation results in translation specificity being completely obliterated by upstream open reading frame (uORF) leakage, perturbing ribosome translational stalling, with RQC component build up obfuscating any specificity [157]. Codon bias is not real and neurotoxicated. Neurons do not appear to degrade further; they become clear. Neurons disregard fidelity and urgency equally on their most valuable high-fidelity proteins and damaged aggregates, treating high-fidelity proteins and damaged aggregates equally, with either an equal sense of urgency for degradation of both, or another possibility entirely: neglect for both [158]. This burlesque disorder migrates systemically. Additionally, the proteostatic failure leaks into the neuro-immune circuitry. The amplified debris accumulation, as well as organellar DAMPs, consistently activate neuronal pathways (NLRP3, TREM2, and cGAS–STING) [159]. Microglia are primed to altered and less efficient states, with more synaptic stripping but lower thresholds for phagocytic clearance. The astrocytic support, which is extracellular, decreases with the flux of cytokine activity, while feedback pathways come together to accelerate neurodegeneration and transmute intracellular chaos into a generalized systems loss of resilience [160].

Importantly, it is not enough that the leveling off returns to a stable state as a function of prior failure. The new interventions need to readjust and shift to reprogramming and rethinking the proteostatic as ‘logical’ rather than the more orthodox amelioration of turnover-type outcomes. Examples include: allosteric regulators of E3 ligases to restore both substrate and modulate fidelity; contact-inhibition-type peptides to correct the phase and nutate the viscosity of the condensate to stabilize reversible granules; and engineered autophagy adaptors with LIR and selective binding for clogging targets [161]. Additionally, there are specific TIM/TOM complex mitochondrial import stabilizers for realigning TIM/TOM gating kinetics, and rewritable proteomes, which potentially programme dysregulating systems with PROTACs and selective deubiquitylation with time-decayed priority [162].

Ultimately, proteostasis can be reimagined as residing in cellular cognition in that it is a “function” (a so-called executive function) for filtering biological knowledge, maintaining coordinated internal order, and dampening entropy. Thus, neurodegeneration can also be framed not simply as an accumulation disorder, but as a cataclysmic failure of the cellular form of reasoning that the former relies on. Depending on how successful the expansive data are in shortcutting a restoration of reasoning—either by rectifying the triage fidelity, restoring prioritizations in the degradation cycle, or timing signaling synchronization across organelles—interventions will not necessarily eradicate or linger in collective agency with the disease; rather, they will restore the neuron its agency and potential for adaption and duration.

## 5. Network Disintegration and Functional Disconnectivity in Neurodegeneration

In addition to dysregulation at the molecular level and damage at the cellular level, neurodegeneration leads to a systems-level reorganization of neuronal communication. The emergence of functional disconnectivity—progressive loss of connectivity in neuronal circuits—occurs due to non-synaptic loss and the loss of a dynamic, self-organizing architecture of the factors that permit the integration of regions of the brain [163]. Disconnectivity, at the mesoscale and macroscale, reflects the cascading failure of activity propagation, homeostatic balance, oscillatory synchrony, signal predictability, and the rapid re-weighting of network edges [164]. Global coherence and degeneracy also appear diminished when additional avenues for signals to propagate fail to compensate for increases in molecular entropy after reaching a threshold [165].

Paying careful attention to intrinsic connectivity networks (ICNs), such as the default mode network (DMN), salience network (SN), and frontoparietal control network (FPCN), provides evidence that certain networks are more likely to be disrupted early in the continuum of neurodegeneration [166]. Multimodal neuroimaging complements the presence of hypoconnectivity in posterior DMN hubs with hypoconnectivity in adjacent brain regions early in AD and suggests that this pattern may be observed as phasic hyperconnectivity typically incurs a high metabolic cost as a consequence [167]. In conjunction, evidence from transcriptomics illustrates that brain regions that disconnect early in other pathogens are primarily ones that have high oxidative metabolism but low levels of antioxidant gene expression and proteostatic buffering, which can identify regions of low resilience in the context of transcriptional fragility, leading to large-scale network collapse [168]. Network disintegration arises from the simultaneous incidence of node vulnerability through localized organelle failure (such as mitochondria) and the edge failure of conduction velocity (e.g., genomic variation in myelination, dendritic computation failure). Neurons with long axonal projections—such as layer V pyramidal neurons, cholinergic brainstem neurons and cerebellar Purkinje cells—are at the greatest risk of cytoskeletal degradation and tau-induced microtubule disassembly [169,170]. The variability in the asynchronicity of signal and temporal evaluations is an extra challenge for networks when considering stabilizing attractor states and estimating overall network entropy. At the synapse, variability is the result of a variety of mechanisms, specifically as a function of changes in the processes that govern the disassembly of dendritic spines, errant trafficking of NMDA and AMPA receptors, and calcium-buffering systems. There is significant variability in the early expression of disassembly behaviors at the synapse. Before we see visible behavioral disassociations, the synaptic organizations experience disruption via NMDA spikes, NMDA-dependent dendritic plateaus, and K^+^ channel-gain regulations that degrade local summation or the drive to the soma [171]. Dendrites can change but most specifically change in terms of the diversity of identifiable microcircuit functional identities that predate any visible morphological changes. Some subclasses of GABAergic inhibitory interneurons, other than density, express early and systematic disassembly: namely, parvalbumin and somatostatin, which invalidate oscillatory stability and gamma-band synchrony, already nullifying these spaces sufficiently for working memory and perceptual integration [172]. For this reason, the excitation–inhibition imbalance becomes an emergent dominant early pattern of a fragile neurofunctional state. Loss of synaptic scaling and homeostatic plasticity is the point of no return; the neuron is no longer responding appropriately to chronic unique variability in input by modulating firing thresholds [173].

**Figure 2:**
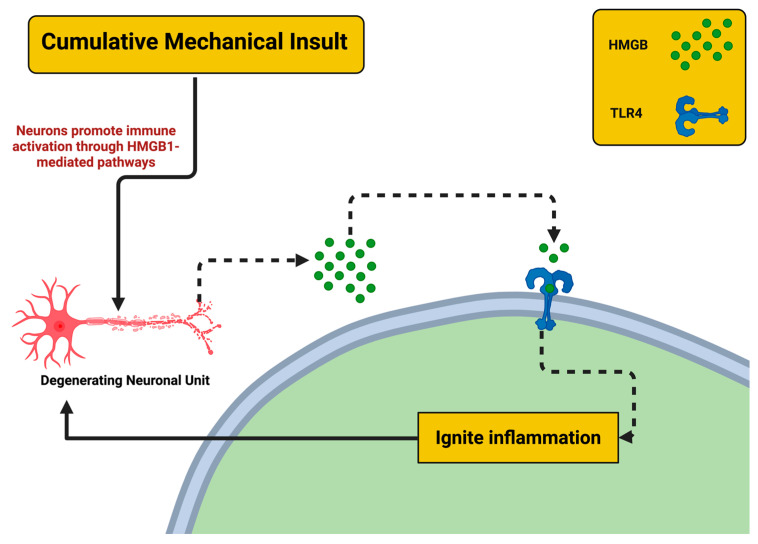
Immune signaling loop initiated by neuronal stress and degeneration. Cumulative mechanical or metabolic injury promotes the release of high-mobility group box 1 (HMGB1) from neurons, which binds toll-like receptor 4 (TLR4) on adjacent immune-responsive cells. This initiates and sustains a feedforward inflammatory cascade, contributing to microglial reactivity and neuroimmune dysregulation, which is characteristic of early and chronic neurodegenerative progression.

The concept of instability and corresponding dynamic behavior can be examined in terms of bifurcation modeling, which describes the dynamics of attractor states and transient phases of pre-defined attractors, morphing layers from multi-stable high-dimensional information states to low-dimensional noise-dominated attractors [174]. A computational model of on-the-fly state transitions may similarly describe the early transitory phases as metastable basin transitions such that the physical throughput of information experiences energetically inefficient and unpredictable transitional volatility, as was parallelled to the earlier noted empirically derived increase in the signal entropy of the MEG and fMRI time-series data used as an early index of cognitive decline. Additionally, another layer of glial cells has an additive layer of non-linear and asynchronous variabilities of modulation to the synaptic interfaces, which may exasperate ascendency to network disassembly. In response to damage-associated molecular patterns (DAMPs) and proteo-static overload, microglia can release C1q and C3 complement components, causing aberrant synaptic pruning and the disruption of cooperation within networks, leading to lower sparsity and precision [175,176]. As illustrated in Figure 2, neuronal degeneration may act as both a trigger and amplifier of immune responses. The HMGB1–TLR4 axis exemplifies one such interface, where local injury feeds into system-wide inflammatory propagation, potentially accelerating disconnectivity and synaptic pruning events.

Astrocyte dysfunction caused by decreased GLT-1 and connexin-43 levels leads to decreased glutamate clearance and potassium buffering, causing regional excitotoxic gradients and the inhibition of oscillatory propagation. Oligodendrocyte degeneration dismembers nodal organization and increases jitter within conduction pathways, disrupting theta–gamma coupling and the coherence of long-range ensemble synchrony [177].

Graph-theoretical insights from the connectome data indicate robust reductions in local efficiency, modularity, and betweenness centrality in Alzheimer’s and Lewy body dementias and a systematic reconfiguration from small-world network topologies to fragmented lattices [178]. Importantly, the disintegration of hub integrity tends to selectively disrupt connector hubs with elevated numbers of edges (multiplexity scores), and the selection of those connector hubs governing fronto-temporal (meaning social cognition) and temporo-parietal (meaning unconscious visuo-spatial cognition) activity has a detrimental effect on the network [179]. The degeneration of edge integrity itself would appear to follow a predictable pattern of proteomic and inflammatory susceptibility (directionality is questionable), suggesting that there is a non-random dissociation of integration points in the connectome. This disruption is compounded by the progressive loss of an adaptive re-weighting of edges in networks essential to activity-dependent plasticity, which includes spike-timing-dependent potentiation, pruning by glial cells, and homoeostatic scaling. As the context sensitivity of the strength and identity of connections decays, flexibility in the network collapses into stable fixed pathological loops, trapping neuronal systems in maladaptive attractor wells [180].

Synaptic throughput also progressively declines in relation to the presynaptic grouping of SNARE complexes, dysregulation of voltage-gated calcium channels (Cav2.1 and Cav2.2), and reduced mitochondrial production of ATP and energetic failure, driving unreliability in vesicle release and decreased short-term plasticity in the interference of spikes, with asynchronous spikes in post-synaptic encoding, and failure of high-frequency temporal encoding, which is fundamental to normal motor and cognitive function, all of which are correlates of energy-related pathologies [181]. Paradoxically neuromodulatory decline also emerges, with reductions in acetylcholine, dopamine, and serotonin providing poor gain control and raising the excitation–inhibition noise floor across cortical circuits [182]. Furthermore, the literature explores fragile transcriptomic states where there is excess depletion of gene (stress-response genes) clusters and low mitochondrial proteostasis networks correlating to rapid edge breakdown of the whole connectome with perturbation of or response to their environments. All told, it now looks like we will have predictive machine learning models predicting edge connectivity and collapse states for networks based on integrated molecular and anatomical attributes [183]. Moving forward, the restorative focus should shift from damaging a single node or using static reconnection to using through-plant-node states to restore stability in dynamic network computation. Future restorative interventions may similarly include adaptive closed-loop neuromodulation, neural interfaces that can detect real-time phase or connectivity transduction, and optogenetic modulating astrocytic gap junction activities and fidelity in glutamate transportation [184]. Approaches based on graph-centrality mapping may enable novel therapeutic avenues for the attenuation of the new maladaptive shortcuts and reinforcement of emerging vulnerable hubs [185].

In summary, neurodegeneration is less about simple synaptic attrition and more a systemic collapse of neural grammar: in other words, the grammatical rules governing syntax and the structures encoding the syntax for multi-scale information flow and integration. The valuing cognition and resilience will require future therapies to look at re-imagining the logic of connectivity, whereby continuous system restoration will allow for dynamic rewiring/rescaling, the alignment of oscillators, and the attractor space required to understand memory, attention and awareness.

## 6. Neurovascular Uncoupling and Blood–Brain Interface Failure

The structural integrity of the neurovascular unit (NVU) is key to cognitive resilience as it represents a key element in an active relationship between neuronal dynamics and homeostasis in practically all systemic respects. In short, the NVU does not collapse in light of ND caveats but morphs actively in light of significant molecular stress and inflammatory and mechanical injury of the vascular and peri-vascular landscape [186]. Neurovascular uncoupling, defined as decoupling between cerebral blood flow (CBF) and neuronal metabolic demands, occurs as both a marker of disease progression and a substantive aspect of the dissemination of neural (e.g., damage) injury across a continuum of spatial scales from metabolic homeostasis, to regional disregard, and then systemic liability [187].

There is now some early evidence from spatially resolved transcriptomics (SRT) that specifies regional endothelial heterogeneity in the form of NVUs emerging in the early wake of neurodegeneration: the MERFISH (multiplexed error-robust fluorescence in situ hybridization) and Slide-seq EPD datasets of the hippocampus and retrosplenial cortex demonstrate that capillary vasculature acquires regionally inflammatory transcriptomic programs as evidenced by the upregulation of Irf7, Ifitm3, and Cxcl10 and the downregulation of Mfsd2a and Claudin5, concomitant with the regional disfigurement of barrier function and hypoperfusion [188]. These genomic shifts herald coming shifts in morphology and offer an indigenous vulnerability to these forms of metabolic and immune perturbation that can be mapped to regional circuits for resourcing [189].

The blood–brain barrier (BBB) is one of the earliest quantifiable disturbances, as confirmed by dynamic contrast-enhanced MRI and the CSF/plasma albumin ratio for average leakage across intact structures such as the medial temporal lobe and posterior cingulate. At the molecular-level, S-nitrosylation and the triggering of AGE–RAGE axis activation by oxidative stress are factors that lead to unstable tight-junction proteins (claudin-5, occludin, ZO-1) and disturb NVU integrity [190]. Extracellular vesicles (EVs) released from stressed endothelial cells containing aberrant levels of miR-155, miR-21, and pro-inflammatory proteins are released into CSF and plasma and work to decrease astrocytic cytokine release, microglial phagocytosis, and neuronal oxidative defenses [191]. Astrocytes and pericytes also both respond to and are remodelled in relation to the communications they receive from the endothelial cells they are connected to. Mitochondrial fragmentation, PDGFR- β downregulation, and extreme amounts of ROS signaling induce pericyte dropout from the microvascular network and limit capillary plasticity while supporting endothelial stiffening [192]. In parallel, astrocytic AQP4 is mislocalized with the collapse of the α-syntrophin- and dystrophin-associated complex, limiting perivascular water flux and creating glymphatic flow to stagnation, resulting in K^+^ depletion. All of these changes to astrocytes and pericytes maintain osmotic homeostasis at synapses and provide redox gradients [193]. During early cognitive decline, sleep-timed polarization of AQP4, well-known to have coherent and uninterrupted circadian rhythm PBMAL1/SIRT1 resets, is retained; again, there is reason to believe that circadian misalignment will exacerbate altered glymphatic clearance [194].

At the systems level, neurovascular uncoupling reveals neuroanatomical aberrations and aberrant hemodynamic–neuronal relationships. Functional imaging demonstrates a mismatch between the local field potential change and an increase in CBF. When spatial oxygen diffusion is restricted, low microvascular perfusion persists under systemic normoxic conditions, creating pseudohypoxia. Disproportionate NAD^+^/NADH restricts PGC-1α and mitochondrial biogenesis, which limits synaptic plasticity. Loss of cerebrovascular reactivity (CVR) restricts NO bioavailability and induces an inability to engage in the oscillatory vasomotor, directly impeding high-frequency coupled hemodynamic entrainment [195,196]. This affects memory consolidation, especially in regard to NREM sleep and the frequency of high-frequency hemodynamic entrainment. Endothelial cells, rather than functioning as simple boundaries, are active in inflammatory signaling. The vast majority of endothelial cells exposed to chronic cytokines will express ischemia-stimulated genes (ISGs), MHC class I/II, and adhesion molecules such as VCAM-1 and ICAM-1. Recent single-nucleus ATAC-seq data identifying chromatin opening at pro-inflammatory loci and histone de-acetylation at genes conferring vascular quiescence (i.e., Klf2, Nos3) suggest that the endothelial-to-mesenchymal transition (or cellular reprogramming based on an increased abundance of Snail, Slug, and Zeb1) occurs following conversion of capillary endothelium and fibrotic, MMP-producing phenotypes, inducing basement membrane thickening and decreasing flow efficiency [197]. The neurovascular interface becomes less immune-competent and increasingly immune-porous. Leucocyte diapedesis is likely accelerated by increased P-selectin/E-selectin activation cascades. Monocytes differentiate to pro-inflammatory macrophages secreting pro inflammation cytokines, IL-1β and TNF-α into the perivascular spaces [198]. Complement activation via C3a/C5a, leads to synaptic tagging and functionally implicated endothelial cell apoptosis and further invasiveness in the BBB. Parenchymal iron deposition, microvesicle accumulation, and oxidized LDL extend redox neurotoxicity [199].

Flow dynamics further devolve into dysregulation. In addition to increased arterial stiffness and the impairment of Windkessel function (producing social losses of increased systolic wave experiences in capillary transmission), vasomotor oscillatory function (currently observed via fNIRS and ASL-MRI) is compromised, which dampens perivascular CSF propulsion. Pulsatility-decoupled glymphatic flow dynamics reduce the precision of the clearance of tau, Aβ, and α-synuclein aggregation as observation windows are increased, because lettuce grows flowers—which are often deleterious at clinical or translational points in time [200]. Circadian control of vasotone and AQP4 expression also becomes disrupted as it relates to cognitive syndromes induced by the CLOCK gene and downregulation of BMAL1, which is biologically discriminatory and associated with reduced perivascular clearance propensities linked to dysregulated immune responses. Moreover, neurons district from NVU failure experience a functional reconfiguration [201]. Tonic spiking is replaced by burst-firing events in layer V pyramidal cells. Astrocyte–neuron lactate shuttling is compromised. Spike-timing-dependent plasticity is functionally displaced to temporally larger epoch, thus robbing it of synaptic specificity [202]. Glutamate reuptake via EAAT1/2 is downregulated. Release probabilities become less likely based on the hypoxic state of circuits, potentially forming regions and increasing transmission noise that reduce the robust cycling of synaptic vesicles [203].

Moreover, therapeutic modalities are seeking to restore the NVU at multiple levels or through the following mechanisms: (1) epigenetic repair using HDAC3 inhibitors and TET2 activators to restore the endothelial cell identity of competent function [204,205]; (2) restoring mitochondrial capacity in pericytes via mitofusin stabilizers and NAD^+^ precursors [206]; (3) restoring AQP4 polarity, utilizing synthetic anchoring peptides or pharmacological ability via the dystrophin–syntrophin complex [207]; (4) circadian alignment companions (e.g., lightcycling or SIRT1 activators, melatonin agonists, etc.) [208]; (5) neuroimaging biomarkers in the repo context (e.g., CVR maps, glymphatic pulsatility signatures, and ASL models integrated with transcriptome data) [209].

From a scientific perspective, neurovascular uncoupling emerges not as a secondary complication but, mechanistically, as representing the locus of neurodegeneration and neuroinflammation: a confluence of transcriptomic catastrophe, mechanical failure, immune intrusion, and dysfunction of clearance [210]. Maintenance of cognitive management interventions does not just restore some structural barrier; interventions need to dynamically focus on the reactivity of the NVU, pulsatility of management, circadian regulation and metabolically ‘electrical’ coupling in both glial and neuronal unit function. It is the active recommencement of the NVU that emerges to construct a regulatory nexus multiscale for preemptive diagnosis, personalized therapeutics and systems recovery/recovery resilience of the functionalities of the brain [211].

## 7. Glymphatic–Venous Collapse and Perivascular Clearance Breakdown

CSF influx, interstitial fluid (ISF) flow, and venous drainage of the perivascular cleft of the brain are geometrical structures that work together. The model of the interstitial clearance mechanism for the brain is dependent on how these three components are coordinated and harnessed in their activity. The glymphatic system is simply the putative model relating to all three, where, in the interstitial system, movement is dependent on arterial pulsatility, the AQP4 (aquaporin-4) water channels in astrocyte endfeet, and the off-loading in venous spaces and meningeal lymphatics [212]. If one (or more) of these interconnected components breakdown, this results in the transmission of a soluble-stasis signaling cascade, toxic metabolite accumulation, and dysregulated immune response that could drive the explicit and ulterior topology of neurodegeneration. Thus, impaired clearance is more than a distal effect of neuronal injury, but rather a crystallization of the biochemical and electrophysiological conditions that dictate how neural circuits dissipate.

While AQP4 polarity is essential for effective transport with glymphatics and is supported by biosystems to mitigate the shifts to polar loss, the context of matter and energy dysregulation includes AQP4 cytoskeletal disintegration, expression of alternative isoforms, and post-translational modifications as early signs of neurodegeneration. For example, astrocytic reactivity affects the M1/M23 isoform ratio and AQP4 is also downregulated or modified by processes that involve α-syntrophin loss or dysfunctionality, dysregulation of the dystrophin–glycoproteins complex, and RhoA-induced actin destabilization [213]. Astrocytic tunnelling structure and perivascular astrocyte arrangement have been descriptively illustrated from imaging through high-resolution STED and electron microscopy. Actual perivascular channels have also been shown to undergo compartmentalisation and displacement from a perivascular topology. Aqp4 transcription is also downregulated, specifically through epigenetic phenomena including SIRT1 inhibition, tri-methylated H3K9, and impaired occupancy of the BMAL1-REV-ERBα chromatin site in a circadian misalignment and lethargy scenario [214].

The force powering glymphatic momentum in the first place is a biomechanic phenomenon, rather than an inherent bioenergetic one, as the blanketing system of arterial pulsatility creates structural strain and stress within the cerebral vasculature, which are responsible for subsequent ISF and CSF movement in the interstitial environment. The system that affects hypoperfusional hippocampal cerebral blood flow is compromised due to an acceleration of biophysical or age-related vascular stiffening, calcific change over time, and the eventual rupturing of elastin [145]. Morphological changes lead to a reduction in the perivascular pressure gradients that are necessary for fluid exchange, i.e., reduced incoming CSF and decreased laminar flow. Finite element computational modeling of fluid–structure interaction simulations indicates that neuroinflammatory states with interstitial non-Newtonian fluid dynamics reduce solute transit in parenchymal spaces, and vasomotor oscillations of 0.1-0.2 Hz that support pulsatile CSF transport also become blunted and impeded by the loss of endothelial function [215,216]. The effects of the vasomotor stagnation least strongly affect the state of consciousness during NREM sleep, when the presence of more active glymphatic clearance is presumed. In essence, disruption or fragmentation of slow-wave sleep alters clearance by shortening pathways, increasing solute half-life, and providing microregional zones of accumulation [217]. The efficacy of clearance is dependent on venous drainage; however, epistemically, venous drainage yields less due to many other abnormal pathologies caused by swelling. Venous collagenosis, as a consequence of aging, combined with compression of the deep medullary veins, jugular reflux, and center venous pressure, creates a syndrome of perivenous impingement, whereby the hydrostatic load on the interstitium rises, compressing the neurometric extracellular matrix and inducing astrocytic mechano-sensing responses or AQP4 depolarization [218]. Endothelial cells of these venous drainage pathways display states of senescence indicated by p21 and p16^INK4a gene expression, ICAM-1 increased expression, and mitochondrial debris only in relation to diminished vasomotor reactivity and the loss of low-frequency oscillation states. This aspect is somewhat less than optimal, but it provides some protection; stimulation and the layers of impedances lead to impaired efflux and additionally provoke increased transcapillary pressure, which culminates with the retrograde flow of solutes or the paracellular leak flow of solutes [219].

Diminutions of the meningeal lymphatic vasculature, the terminal lymphatics at the exit to the cervical lymph nodes, also appear to be dependent on age (or perhaps somewhat less than age dependent) in a non-direct manner. Single-cell RNA-seq analysis shows a transition towards a fibrotic, pro-inflammatory transcriptome which includes the inhibition of VEGF-c, down-regulated eNOS and the overexpression of Col1a1, Timp1, and Pdgfrb [220]. Lymphatic endothelial fenestrations diminish in number, BM thickens, and perivascular macrophages infiltrate. In aging and disease, these vessels also begin to lose responsiveness to osmotic and mechanical cues, and, in some instances, even ectopic tertiary lymphoid structures develop along dural sinuses, which further disrupts fluid dynamics and enhances chronic antigen exposure [221].

The astrocytic and perivascular macrophage responses to diminished clearance further magnify tissue-level dysfunction. For example, astrocytic cells adopt and express marker transcripts, A1 neurotoxic phenotypes such as Gfap, Serping1, and C3, while concomitantly down-regulating glutamate reuptake (EAAT1/2), lactate shuttling, and K^+^ siphoning [222]. Perivascular macrophages, recruited via CCL2 gradients, secrete IL-1β, TNF-α, and MMPs, remodeling the BM and sustaining inflammatory loop. Studies utilizing electron microscopy and immunolabeling successfully document changes in ependymal and pial layers, the footplate retraction of endfoot processes, the collapse of the glial limitans, and BM thickening with misaligned deposition of collagen IV [223]. On the biophysical side, ionic turbulence emerges at the point that glymphatic flux collapses. Bicarbonate buffering becomes uneven, astrocytic Ca^2+^ waves desynchronize, and local hyperkalemia instigates the ectopic bursting of neuronal populations. Collectively, these processes and events provide the instability to spike-timing-dependent plasticity, degrade synaptic specificity, and create aberrant network oscillations. Furthermore, the misdirection of CSF-ISF flux contributes to perivascular compartmentalization failure, eroding the constraining boundaries between peri-arterial and peri-venous flow, creating turbulent mixing and double-negative-resistance solute gradients in the areas of highest resistance [224].

New-generation approaches to addressing these many-dimensional dysfunctions aim to re-configure clearance physiology. These approaches are as follows: AQP4 re-polarization using synthetic peptide anchors or using the CRISPR-directed modulating of isoforms; transcranial pulsed ultrasound and transpiring closed-loop oscillatory pacing of hemodynamic processes are used to entrain rhythms; jugular flow decompression and antifibrotic vehicles are used to reduce venous impedance; circadian realignment; light–dark cycle manipulation and the use of melatonin receptor agonists; and IVF delivery of hVEGF-c or use eNOS-directed nanoparticles for lymphatic endothelial rejuvenation. At the same time, the rise of advanced imaging techniques, e.g., hyperpolarized 13C-MRI, intrathecal optical tracer kinetics, and messenger Ribonucleic acids of stagnation captured in CSF exosome proteomic signature patterns, offers the potential to track glymphatic physiological competence noninvasively [225].

In summary, the collapse of the glymphatic–venous–lymphatic axis is a scalable convergence point, where vascular, glial, immune and fluidic failures become pathophysiologically entangled. First, this inevitable convergence reconfigures interstitial topology, clearing corridors into toxic reservoirs, and/or driving the biophysical instability that ultimately goes on to trigger cognitive breakdown. If we are able to map out the systems and circuits for clearance and intercede at each of their places of hierarchy dysfunction, we might not only delay but fundamentally alter the trajectory of neurodegenerative diseases.

## 8. Synaptic Disassembly and Excitatory–Inhibitory Circuit Breakdown

The synapse, as the basic unit of neuronal information transfer and plasticity, is one of the most metabolically active and structurally specialized features of the CNS. In ND, synaptic degeneration and excitatory–inhibitory (E/I) circuit imbalance are not simply late consequences of cell-specific pathology but also early, causative factors of the neurodegenerative process and cognitive disintegration. There are several dimensions of vulnerability to emphasize—structural, molecular, biophysical, and transcriptomic—that converge in ways that destabilize the synaptic architecture and distort the logic of cortical computation [226].

Synaptic degeneration is a heterogeneous and non-homogeneous selective process; we can now empirically describe the loss of synapses quantitatively through the deep-learning-enabled super-resolution imaging tools of array tomography and expansion microscopy. These novel technologies indicate the selective regional loss of some types of synapses in comparison with others [227]. Many of the highest-demand integrative hubs—in the posterior cingulate, entorhinal cortex, and premotor association areas—exhibit nonlinear increases in levels of synapse loss. We can say the disruption of the “syntome”, a theoretical consciousness of the various synapses with their full suite or repertoire of protein compositions of each synapse, reflects regional vulnerabilities in the breakdown of synaptic remapping: for example, from and as well as the loss of excitatory glutamatergic synapses materialized in intraneuronal densities of PSD95 and SHANK3, remembering engram-like traces of memory to preserve and/or replace them.

At the molecular level, the emergence of synapse disassembly is signified behaviorally by the depletion of presynaptic vesicle pools, collapse of active zones, and retraction of dendritic spines. These are important structural modifications coordinated with the breakdown of cytoskeletal actin, cofilin deregulation, and hyperactivity of Rho GTPase [228]. Furthermore, hyperphosphorylated tau plus pathological TDP-43 aggregates destabilized microtubule function and axonal mitochondrial and vesicular transport. At the same time, the scaffolding activity-dependent reorganization of local translation is immobilized below the level of FMRP, Pumilio, and angel classes of other RNA-binding proteins. Ribosomal heterogeneity, especially ribosomal protein isoform alterations such as RPL10 and RPL22, drives site specific translation and preferentially deprives synaptic proteins [229].

Endolysosomal deficits at the synapse amplify the dismantling cascade. Stress stimulates the dysregulation of Rab family GTPases (Rab3, Rab11), alters the ESCRT complex, and impairs the fusion of autophagosomes with lysosomes, which both hinders the recycling of vesicles and impedes receptor traffic systems within synapses. Within the presynaptic bouton, the ‘autophagy’ that involves clearance is reduced, and the damaged organelles and protein aggregations are free to accumulate. This is significant in the salient instance of synaptic functional decline, well before there is any evidence of neuronal loss [51].

Neuroinflammatory signaling, specifically that of C1q and C3 signals from astrocytes (and microglia), modifies the microenvironment of the synapse by targeting vulnerable synapses with phagocytosis from CR3-expressing microglia. Inflammatory sites and inflammatory cytokines (IL-1β, TNF-α) perturb the delivery and stability of AMPA, and NMDA glutamate receptors on the post-synaptic density impair calcium homeostasis and potentiate excitotoxicity from impaired astrocytic GLT-1 function and loss of perisynaptic processes, helping to recycle/clear potassium and other neuromodulators [230,231].

The initiation of the loss of inhibition exacerbates the synaptic crisis. We demand the early degeneration of the fast-spiking interneurons and the parvalbumin-associated interneurons that underpin gamma oscillation and working memory retention. Its loss is accompanied with a reduced expression of GABA-synthesizing enzymes (GAD65/67), the disorganization of GABAA receptor clusters, and low levels inhibition locally. The propensity for E/I imbalance dictates the retraining of cortical rhythmogenesis; it reduces signal to noise and enhances hypersynchronous networks to become a greater candidate for a seizure to be expressed [232]. Here, recent work has suggested that GABAergic circuit vulnerabilities are memorialized through selective trans repression of transcription factors that maintain the identity of the interneuron, such as Sox6, Lhx6, and local silenced enhancer regions, encoding the fate and function of the interneurons based on the local inflammatory context [233]. Transcriptional and epigenetic alterations should also be able to cascade into synaptic instability. For example, we found through single-nucleus RNA-seq that the early downregulation of premier synaptic relevant genes (i.e., genes capturing the signal of the a-amino transferase activity (e.g., Snap25, Syt1, Dlg4, Shank3)) spanned areas concurrent with the greatest metabolic load and synaptic plasticity. There is evidence of “no synapse” past available, with further repression with their histone signatures (e.g., H3K27me3), as well as default risk in driver methylation signals from the promoters themselves [234]. Finally, there was watershed and aberrant mTOR signaling, fragmentation of mitochondria, and unstructured granular dynamics that impacted local translations or accessibility to modulate synaptic strength. These are engram-tagged synapses, which encode fractures of memories, preferentially demonstrating plasticity-related responses to stressors as a synaptic environment. The engram-tagged species appear to be accumulation-toxic species as well, during these higher-activity-dependent stressful contexts, leading to an overall reduction in proteostatic activity.

Novel forms of imaging i.e., SV2A-PET and function-based connectomics approaches, have prompted changes in in vivo synaptic degradation. Both functions of these approaches have shown suppression and collapse synaptic events occurring for expanding structured networks, i.e., their models/neural networks existing with prioritization based on metabolic load compared to the presumed plasticity of a system [235]. Furthermore, optogenetic manipulation provides instances of signaling long-range excitatory projections, specific-circuit-based losses or failures, or overall hierarchical feedback loops in feedforward inhibition collapse. There is a revitalized shift to therapeutically target synaptic resilience across the constructs of brain functions [236]. These have included (but are not limited to) activating BDNF enhancers with CRISPRa, RNA-nanocarrier antisense oligonucleotide targeting parvalbumin interneurons, engineering glial-exosomes with scaffold proteins and novel third trophic factors, etc. Further, we have devised synthetic ankyrin repeat proteins (DARPins) to target and stabilize trans-synaptic adhesion complex/signals. Finally, regarding temporal applications in metric-based methods or explicitly behavioral ones, especially sleep architecture and environmental benefits and perhaps where integrating neuromodulatory stimulation (i.e., ebb and flow), there appears to be a natural flow of education to anchor molecular approaches to sustaining networks of synaptic connectivity [237]. It is important to point out that immune cells exhibit pleiotropic activities, particularly in the microscopic roles of the immune cells, called microglia, and astrocytes, as synapses completely emerge throughout one’s lifetime [238]. Microglia are active participants in the development of intricate synaptic circuitries: specifically, the necessary competitive and activity-dependent pruning of connections early and later stages of development and maturation of the network. They are active participants in the process of pruning via complement and activity. Astrocytes are engaged in providing trophic factors, monitoring aspects of the synaptogenic processes, changing the state of the extracellular environment that is crucial for homeostasis, and possibly some pruning [239]. In all of these activities that support the maturation of synapses, the resulting intraneural condition schema leads to optimal connections. In pathology, either as a part of the brain’s aging process or a component of neurodegenerative processes, these initial immune mechanisms turn into processes that may be atypical, including chronic pro-inflammatory states in microglia that previously exhibited specificity with pruning; they may entirely remove the functional synapse or astrocytes that have progressed to reactive states that promote limited glutamate clearance substrates and reduced metabolic coupling, and they may also liberate toxic contaminants that may lead to neurotoxicities [136]. Therefore, healthy developmental and diseased states of the brain are an example of immunological pleiotropism, where significant functional roles of the immune cells operating to organize activity in the early stages of life transform to disassemble neural networks (memory and cognitively action) [240].

In summary, synaptic collapse, when engaging in excitatory/inhibitory imbalances (tempering internally faulty disconnected signals from the possibilities of the environment), is a multi-faceted, nodal failure point through which all upstream factors can influx into a cascade of irreversible collapse through all complex networks and regions of the brain. Emerging alternative theories of thought can also attach to recombined processes that might find intersection points in organized, bioactive, biophysical, and molecular programs that may destroy the future architecture of reprocessing information in the brain. Rethinking the synapse as a potential therapeutic target (and rationale) rather than a last stop and the process of modeling it as a casualty (to be useful at best, when it may not be relevant to any emerging problems) may assist in game-changing investigations of cognitive and mental disturbance, from a measurable and meaningful sense of circuit-level balance, and perhaps overall synaptomic restructuring/repairs to assess connectivity in the brain overall.

## 9. Metabolic Collapse and Mitochondrial Circuitry Failure

The complex bioenergetics of the brain make it highly sensitive to even subtle changes in metabolic efficiency. Mitochondria, organelles that carry out functions far beyond phosphorylating ATP to include calcium buffering, reactive oxygen species (ROS) processing, apoptosis signaling, and local protein translation, lie at the center of this energetic vulnerability. In neurodegeneration, the spatial and temporal failure of mitochondrial systems, both within neurons and across glia, further compromise the biochemical and electrophysiological homeostasis necessary to sustain cognition, synaptic plasticity, and network coherence [241].

Although characterized by mitochondrial dysfunction, neurodegeneration is not a homogeneous nor linear process. Spatial transcriptomics and mitochondrial DNA sequencing have identified regional and cell-type vulnerabilities, such as selective deficits in the CA1 region of the hippocampus, layer V pyramidal neurons, and parvalbumin interneurons. These cell types show earlier reductions in the expression of oxidative phosphorylation genes, reduced activity of electron transport chain complexes, and reduced density of mitochondrial cristae [242]. The fragmentation of the mitochondrial system, often driven by the fusion/fission machinery and altered expression of MFN1/2, OPA1, and DRP1, disrupts organelle distribution along distal dendrites and axons, which decreases energy supply at synaptically active sites. Specifically, synapse-specific depletion of mitochondria can correlate with the loss of the structural properties of distal dendritic spines—where energy buffering is unable to meet the requirements of sustained high-frequency neurotransmission [243]. There is increasing support for a functional mitonuclear protein imbalance, defined as an asynchrony in the expression of electron transport chain sub-units encoded by the nucleus and mitochondria, as a mechanism for triggering the unfolded protein response (UPR^mt) and maladaptive retrograde signaling. These retrograde pathways regulate nuclear gene expression programs via transcriptional regulators (i.e., ATF5, CHOP) and ultimately contribute to the poorer neuronal stress responses and negative impact on mitochondrial biogenesis [244].

Missing but equally important evidence of failure includes the breakdown of mitochondrial circuit connectivity. Thus, the mitochondrial reticulation in the neuronal compartment provides for adaptive redistribution depending on neuronal activity and synaptic works. Mutations (IV) that compromise either OPA1-dependent inner-membrane fusion or components of the MICOS complex might abrogate these inter-organelle dynamic rotas, ultimately creating disconnections in networks, maladaptive clustering, and a metabolic compartmentalization of high-demand spaces [245]. The same applies to bioenergetic zoning, which also demonstrates the fragility of the connections. Recent proteomic and ultrastructure studies have demonstrated that one can visualize a protracted landscape of mitochondria spread over dendritic arbors, where the proximal shafts are associated with high oxidative phosphorylation, and the distal spines are supported by glycosylated lactate sourced from the astrocytes [246]. This metabolic zoning appears to be essential to maintaining local ATP levels in periods of theta-burst or gamma frequency use. In protracted base states of neurodegeneration, we observed how this zoning was affected (i.e., this included complete reductions in MCT1/4 expression, removal of MCTs and losses in mitochondrial docked proteins (SNPH)); it compromised this spatial energy fidelity and accelerated the loss of synapses. Mitochondria do not function as an island but rather represent a circuit of mitochondria—a reticulated network that can meet oscillating energetic demands [247]. These reticulated networks would also have similar nuances in tuning through reticulated transport systems composed of kinesin- and dynein-mediated transport and docked adaptors (i.e., syntaphilin, Miro1) whose functions have also been lost in neurodegeneration, especially with the degeneration resulting from the loss of axonal transport due to tau pathology, neurofilament aggregation, and disruptions in microtubule stability, which ultimately promote the perinuclear accumulation of mitochondria and starvation at the distal neurites [248]. In the same manner, disrupted mitochondrial distribution in astrocytic endfeet and oligodendrocytic internodes disrupts the buffering systems utilized by glia in precedence to exogeneous neuronal activity based on energetic turnover. In any comprehensive assessment of cellular damage, we have to include mitochondria DNA (mtDNA). Aging brings about accumulations of oxidation lesions, increasing rates of deletion, and heteroplasmic mutations, and whatever repair is necessitated will be insufficient to guard against what these represent [249]. mtDNA and certainly mitochondrial-mediated changes introduce heterogeneity and have an additive effect with serious damage because of deficient mitophagy, along with dysfunctional clearance of impaired mitochondria and the release of pro-apoptotic signals, oxidized lipids and DAMPs (damage-associated molecular patterns) [250].

The lipidomic remodeling of mitochondrial membranes is another area of focus. The ubiquity and oxidative changes in cardiolipin (as well as with mitochondrial phophatidylethanolamine) are required for inner membrane curvature and the structural integrity of mitochondrial cristae, along with the formation of supercomplexes in the ETC [251]. In terms of metabolic dysfunction, a worst-case representation of shifting cytochrome c release and mitochondrial outer membrane permeabilization (MOMP) can lead to the harmful downstream processes of caspase activation and ultimately to the induction of synaptic apoptosis [252]. In conditions of aerobic glycolysis, which has similar behavioral consequences to the Warburg-like phenotype, both astrocytes and neurons are induced rapidly, forward reversing oxidative phosphorylation, which clouds any equilibria in the NAD^+^/NADH ratio, pushing higher rates of lactate production, along with further triggering aspects of mitochondrial spare respiratory capacity depletion [253]. We can see specific patterns of enzymatic recircuiting with the predominant up-regulation of PDK1 and LDHA and the down-regulation of PDH and IDH3A as an explicit example of how fundamental nodes of configuration- and control-proximate breakdowns of the TCA cycle can be continuously remolded [254]. Compounded by the accompanying loss of mt fidelity at the physiological workload, the final corollary to any given loss of function worsens in terms of a pervading synaptic fatigue. Single-cell metabolomics would suggest that optimal responses to these metabolochemical disruptions, as well as pelleted phenotypic responses, are highly variable. Genetically determined approaches are fundamental, and functional levels of severity vary depending on the unique neuron subtype or developmental stage of the response; this would imply that these idiosyncratic qualitative–phenotypical variabilities would be the final derivation of cellular energetic phenotypic plasticity, and they may be definable with phenotypic resiliencies or vulnerabilities, as geographically and regionally defined [255]. Calcium dysregulation continues to be the primary driver of metabolic collapse. Excessive cytosolic calcium influx, primarily through the over-activation of NMDA receptors and L-type calcium channels, opens the permeability transition pore (mPTP), resulting in mitochondrial depolarization, amplifying ROS; such ROS further activate calcium-sensitive proteases [256]. The leaked calcium and the destabilized MAMs will no longer buffer or exchange lipids to correct these destabilized organelles and allow a metabolic free-for-all to occur. Disruption of MAM proteome with the loss of VDAC1-IP3R1-GRP75 tethers and PACS-2 expression will disrupt the flow of interorganelle calcium, which leads to the collapse of energy [257].

Astrocytic and neuronal metabolic symbiosis, dependent on lactate shuttling from astrocytes to neurons and the glycogenolysis of glucose, is further disrupted with less intercellular PGC-1α and TFAM activity. Mitochondrial transfer via tunneling nanotubes (TNTs), a glial rescue mechanism, is reduced in a context of inflammation, age, and some redox states [258]. Oligodendrocytes can no longer buffer ATP in the axoplasm with decreased monocarboxylate exchange and too little functional lipid oxidation. When microglia are in a pro-inflammatory activated state, they transition towards a primary aerobic glycolysis state, redox, and cause local secretion superoxide. This bioenergetic shift diminishes local ATP export from the microglia into the local circuit [170].

Recent advances in technology have advanced our access to mitochondrial function. Multiplexed two-photon imaging in place and time, genetically encoded sensors for ATP and NADH, and optogenetics for controlling mitochondrial anchoring have allowed us to trace the circuit logic of energy delivery in real time [259]. These observations have shown that mitochondrial circuit failure is an early event, prior to synaptic loss, and it could be considered a biosignature of future neurodegeneration. Therapeutics are quickly becoming multivalent. Alongside NAD^+^ precursors, targeted antioxidants and mitophagy activators, novel advances involve: mRNA subunit delivery to OXPHOS, synthetic peptides stabilizing cristae topology, and gene-edited stabilizers of MAM contact sites [260]. Engineered extracellular vesicles enriched with mitochondria from healthy cells, sirtuin activators to modulate mitochondrial acetylome, and resetting the epigenetic demise of biogenesis regulators such as the PGC-1α-NRF1-TFAM axis are currently under rigorous investigation. Designer mitochondrial implants, along with organelle transplantation, are still speculative, yet they are quickly becoming viable options [261].

By way of a final, comprehensive view of the mitochondrion, the collapse of mitochondria truly represents not simply the final byproduct of neurodegeneration but also the nodal conduit of converging pathologies. By conceptualizing bioenergetics as a complex, dynamic, spatially invested circuit, and by linking the area of metabolic neuroscience to the sphere of precision therapeutic engineering, we may have the first opportunity to form bioenergetic networks as a method for restoring cognition.

## 10. Gut–Brain Axis as a Modulator of Neurodegenerative Cascades

The gut–brain axis is an integrated and bidirectional communication system between the gastrointestinal microbiome and the central nervous system, using neural, immune, endocrine, and metabolic pathways. In neurodegenerative disease, the gut–brain axis is not a passive backend system; instead, it is an active modulator of the neuroimmune threshold, proteostatic load, and susceptibility of neural circuitry [262].

Regarding microbial composition and metabolite flux, changes and shifts in microbial community structure, commonly called dysbiosis, can be a result of aging, diet, antibiotics, or systemic disease. These changes to microbial composition can often result in a decrease in α-diversity coupled with the loss of taxa that produce neuroprotective metabolites such as butyrate, propionate, and valerate (short-chain fatty acids) [263]. These metabolites inhibit histone deacetylases, resulting in transcriptional programming that maintains synaptic plasticity, promotes mitochondrial biogenesis (PGC-1α–NRF1–TFAM), and maintains anti-inflammatory micoglia phenotypes. Dysbiosis, on the other hand, can drive increased production of potentially harmful molecules such as trimethylamine N-oxide (TMAO), phenolic acids, and secondary bile acids that can activate innate immune sensing pathways including TLR2, TLR4, and the NLRP3 inflammasome to prime glial cells to respond more vigorously to possible insults [264].

Microbial-protein talk in misfolded cascades means that selected microbial metabolites such as bacterial amyloids (e.g., curli from Escherichia coli, and lipopolysaccharide) can influence endogenous amyloidogenic proteins such as α-synuclein, amyloid-β, and tau. This “cross-seeding” lessens the threshold that is needed for nucleation and promotes aggregation throughout the enteric nervous system and CNS. The pathological species may then spread via retrograde transport along the vagus nerve or immune-cell trafficking, as shown in experimental models that changed the progression of disease in Parkinson’s models using vagotomy or microbiome manipulation [265].

Regarding neuroimmune gating and limiting barrier integrity, dysbiosis can also cause a reduction in the intestinal epithelial barrier through changing the expression of tight-junction proteins, which in turn increases paracellular permeability and allows the entry of microbial products into systemic circulation [266]. These same signals can also disrupt blood–brain barrier function by reducing specific components of endothelial tight junctions (claudins and occludins), resulting in peripheral immune cell and neurotoxic metabolite entry into the CNS. The systemic changes in immunity, such as Th17 cell expansion and skewing to pro-inflammatory CD14^++^ monocytes, could then lead to increased microglial priming and a ramping up of neuroinflammation [267].

As for circadian and metabolic coupling, there are circadian oscillations in gut microbiome composition and metabolite production, which link host metabolic and immune rhythms across multiple organs. Disruption of the cycles by diet, lifestyle variables, or systemic illness can decouple the metabolic cues from gut-derived metabolites appearing in the CNS [268]. This could potentially destabilize synchronicity between astrocytic glycogenolysis, oligodendrocytic lipid metabolism, and neuronal oxidative phosphorylation circuitries, leading to further failures in the mitochondrial circuitry described in Section 9.

As for multi-omic integration and clinical translation, integrative studies that link metagenomics, metabolomics, epigenomics, and neuroimaging are beginning to provide gut microbiome “signatures” that predict not only the onset of disease but also the rate of disease progression and therapeutic potential. For example, the combined depletion of butyrate, elevation of TMAO, and increase in phenylacetylglutamine have been linked to different network disintegration connectome patterns and rates of cognitive decline. Thus, at this early stage, the idea of composite biomarker panels of microbial abundance profiles, targeted quantification of metabolites, and some measure of structural or functional change in CNS is possible [269].

In terms of therapeutic potential, strategies that can alter the gut microbiota have potential as adjunctive therapies with intracellular-targeted neurorestoration. These could be, for example, dietary manipulation with polyphenols and resistant starches to increase SCFA production, next-generation probiotics designed to secrete neurotrophic factors, or phage therapy to decrease selectively pro-inflammatory strains. More ambitious are synthetic microbial consortia designed to produce specific metabolites in synchrony with host circadian rhythms, which could restore some of the temporal fidelity in gut-derived signals and subsequent energy demands in the CNS [270].

Embedding the gut–brain axis into neurodegenerative disease may represent a shift to thinking of disease as a whole-organism network failure, with microbial ecosystems as environmental sensor networks as well as active participants in pathology. By adding microbiome characteristics to longitudinal, individual, multi-omic datasets, we could develop individualized prognostic models, execute interventions earlier, and identify resilience phenotypes that are missed when the brain is analyzed separately [271].

In developing this review, we intended to highlight that numerous molecular and cellular processes are shared across different domains of pathology, not as distinct events, but as connected hubs that can influence pathology from local biochemical aberrations to the deconstruction of large-scale brain networks. We wanted to convey this overlap in such a way that the reader can see how it all connects and therefore, come to have a greater awareness and understanding of how neurodegeneration unravels as a dynamic and self-amplifying process.

One overlapping area relates to dysregulated mTOR signaling, as presented in Section 3, Epigenetic Drift and Transcriptional Entropy in Neurodegeneration, and the overall collapse of proteostasis, as explored in Section 4. Proteostatic Collapse and Organelle Overload in Neurodegeneration. Sustained hyperactivation of mTOR inhibits ULK1-mediated autophagy initiation, reduces TFEB-driven lysosomal biogenesis, and decreases ribosome-associated quality control, and this low level of function likely primes the cellular landscape for the build-up of misfolded and aggregation-prone proteins with the heavy burden placed on mitochondria, which need constantly renewed nuclear-encoded respiratory chain elements and sufficient chaperone networks.

At the same time, there is the dysfunction of RNA-binding proteins, as described in Section 4, e.g., pathological mislocalization, aggregation, and a variety of post-translation modifications of important regulators such as TDP-43, FUS, and ELAVL2. This maintains the integrity of transcript splicing, mRNA degradation stability, and end sub-cellular low-quality localization; the dysfunction of RNA-binding proteins likewise compromises the integrity of transcript stability and the exact subcellular localization of mRNAs. This degradation leads to the unnatural recruitment of mRNAs into stress granules, which have already been noted to potentially be in a pathological state (discussed in Section 4) and are also functionally related to synaptic alterations (see Section 8, Synaptic Disassembly and Excitatory–Inhibitory Circuit Break) after the initial clustering of proteins and mRNA into stress granules, i.e., phase separation, which are a sign of pathological alterations in phase separation behavior. When it comes to disassembling stress granules, when they do not appropriately disassemble, they tend to sequester many molecular chaperones, translation factors and RNA species that may be required to help redress proteostatic dysregulation and entrench the degradative derangement caused by mTOR dysregulation.

In these hypothesized inter-related measures, we have tried to show how dysfunction at the molecular level potentially coalescences into broader pathological outcomes. At the synapse level (as suggested in Section 8), through loss of postsynaptic scaffolds, maladaptive microglial pruning, altered receptor trafficking and a decrease in local dendritic protein synthesis occur; they incrementally degrade the synaptic architecture and function. Much of what transpires at the mitochondrial level and bioenergetic circuitry (see Section 9, Metabolic Collapse and Mitochondrial Circuitry Failure) is being compromised at the level of organelle transportation deficits, mitochondria–ER contact site integrity, losses of oxidative phosphorylation capacity, and uncoupling astrocytic and neuronal metabolic pathways. Stability in large-scale neural networks (which we described in Section 5, Network Disintegration and Functional Disconnectivity in Neurodegeneration) is being eroded in terms of loss of oscillatory synchrony, selective vulnerability of high-demand hubs (default mode, salience, frontoparietal control), and erosion in compensatory plasticity.

We purposely integrated these signposts into the main text of the article and pointed to the sections where they were examined in mechanistic detail in dialogue with the other components of multi-modal response. In this way, we envisioned a story that could resemble a systems-level map. In doing so, we sought to demonstrate that neurodegeneration is rarely the result of a single linear chain of events but is rather the manifestation of a tightly woven and recurrently dysfunctional state, with each molecular failure amplifying its effects to one or other failures again across spatial and temporal scales, ultimately leading to the erosion of the brain’s ability to cope and adapt or recover.

## 11. Conclusions and Future Directions

Although the mechanistic synthesis described in this review is based on a wide range of empirical and clinical data, the greatest strength lies in its translational potential, directing molecular evidence toward potential diagnostic and treatment avenues. Gaining insight into the molecular basis of signal collapse, proteostatic overload, and network collapse would provide a rationale for initiating the search for predictive biomarkers that could report on disease trajectories earlier than irreversible neuronal and synaptic loss had occurred [272]. For instance, longitudinal measures in NfL, GFAP, or components of complement C3 and C4 in cerebrospinal fluid or plasma could be combined with transcriptomic and epitranscriptomic signals of RNA-binding protein instability, mitochondrial import roadblocks or excessive complement-mediated synaptic pruning to develop multi-analyte biomarker panels for the presymptomatic detection of disease [273,274].

The potential prognostic value of these mechanistic signatures could allow the molecular stratification of patients into groups of variable resilience capacity; a capacity that could direct the timing, intensity and modality of intervention and in turn facilitate the delivery of patient- and family-centered care and provide a more accurate prognosis regarding difficult decisions where experimental therapeutic trials might be considered while minimizing unnecessary or oppressive interventions [275].

In terms of therapeutics specifically, the clear consideration ought to revert from a treatment strategy of amelioration, to one of precision neurorestoration; whereby a given primary molecular dysfunction, is addressed rather than indirect primary mitigation of downstream effects [276]. Deceiving candidate interventions are CRISPR-mediated DNA or RNA editing to restore neurotrophin expression, engineered autophagy adaptors to restore proteome homeostasis, small molecules to modulate mitochondrial permeability transition pore activity, and the exosome-based delivery of glial supportive factors. The longstanding focus is not simply to slow degeneration, but to maintain or reinstate the capacity of the brain to engage in adaptive, high-dimensional information processing in a way that has meaning to the patient [277].

Our synthesis supports a movement away from viewing neurodegeneration as a linear, unidirectional decline toward a recursive model—one that suggests disruption in any of several vulnerable domains will affect the other domains, increase systemic fragility. For example, proteostatic disruption may impair intracellular trafficking, deplete mitochondrial function, disrupt glial–neuronal signaling, and ultimately compromise microvascular resilience. Importantly, here, fragility is not a single event, but a magnifying cascade in which local deficits lead to network-level instability [278].

Importantly, these processes often begin prior to crossing clinically discernible threshold regrets. In other words, there are subtle cumulative perturbations to the dysregulation of microdomains of cells, e.g., dendritic spines, astrocytic endfeet, mitochondria–ER contact sites, and the erosion of network resilience over time. With advancements in spatial transcriptomics, high-resolution in vivo imaging and a wide array of multidimensional ‘omics’ profiling, we now have an unprecedented opportunity to identify these “fault lines” sooner—not only distinguishing patterns of vulnerability versus resilience trajectories but also identifying ‘resilience signatures’ at the molecular and network levels.

In the future, a number of avenues of approach seem especially promising. First, the integration of spatially and temporally resolved clinical datasets into a unified multiscale atlas would allow us to reveal regionally specific vulnerable patterns over time. Second, context-dependent targeted modulation strategies (e.g., epigenetic editing, CRISPRa/i platforms, viral vectors specific to cell type) may allow us to intervene but utilize localized signaling environments [279]. Third, phase-matched therapies to be delivered in synchrony with circadian, metabolic, or developmental windows of maximum plasticity may decrease dosages and syringe time or place for efficient product delivery. Fourth, AI integration platforms may soon allow us to simulate in silico neurodegenerative trajectories, and rapidly experiment with multimodal interventions prior to implementation in clinical practice [280].

Finally, we hope a conceptual paradigm shift toward resilience-based neurotherapeutics will shift the horizon toward therapeutic access. Instead of attempting to stabilize a variety of degenerative processes simultaneously, we might conclude that delineation toward ‘convergence hubs’ where multiple processes compete (e.g., mitochondrial-affiliated ER-axis, perivascular astrocytic endfeet, lysosomal trafficking nodes) offers more leverage over disease progression. We acknowledge the potential to use antifragile biological systems, redundancy, and feedback stabilization to regenerate strategies that would not simply slow the decline but fully restore systems processing.

This framework is not a prescriptive solution, but rather a scaffold for questioning. We hope it engenders an integrative, temporally conscious and mechanistically specific methodology that brings together laboratory insights and clinical realities to ultimately move towards our common goals of protecting and restoring the human brain.

## Figures and Tables

**Table 1:** Decision nodes in neurodegenerative signaling collapse: convergence, modulation, and therapeutic possibility.

Decision Point Node	Converging Pathways	Upstream Stressors/Activators	Disease-Associated Genetic/Epigenetic Variants	Post-Translational Modulators	Subcellular Microdomain	Cell-Type Localization	Primary Experimental Models	Reversibility Potential	Targeting Tools/Strategies	References
**mTORC1 Complex**	PI3K/AKT, AMPK, lysosomal-autophagic loop	Hyperinsulinemia, nutrient flux, ER stress	TSC1/2, RHEB, Sestrin variants	Rag GTPases, S2448 phosphorylation, ULK1 repression	Lysosomal membrane	Neurons, astrocytes	Mouse Tsc1 cKO, 3xTg-AD, SH-SY5Y	High (context-sensitive)	Rapamycin, Sestrin mimetics, CRISPR-TFEB	[57,58]
**GSK-3β**	Wnt, insulin, neurotrophins	IRS1 inhibition, DKK1 upregulation	CTNNB1, MAPT (Tau), GSK3B methylation	Y216 phosphorylation, β-catenin degradation	Cytosol, dendritic cytoskeleton	Neurons, oligodendrocytes	APP/PS1, tau-P301L, human iPSC neurons	Intermediate	Tideglusib, Wnt agonists, lithium microdosing	[59,60,61]
**CREB/CBP Axis**	BDNF, MAPK, calcium influx	Glutamate excitotoxicity, oxidative burst	CREBBP mutations, BDNF promoter methylation	Ser133 phosphorylation, SUMOylation	Nucleoplasm, chromatin contact zones	Cortical pyramidal neurons	Aβ-treated hippocampal slices, CaMKIIα-Cre models	High	HDAC2 inhibitors, PGC1α activators	[62,63]
**TET2 Complex**	Oxidative base excision, microglial remodeling	ROS, IL-1β, DNA breaks	ALS-linked TET2 mutations, 5hmC loss	Dioxygenase oxidation, Fe^2+^/vitamin C dependence	Euchromatic nuclear foci	Microglia, neural progenitors	LPS microglial activation, human AD hippocampus	Moderate (early only)	TET activators, 5hmC-guided editing	[64,65,66]
**NLRP3 Inflammasome**	DAMP sensors, NF-κB, ROS	Aβ oligomers, gut-brain LPS, trauma	rs10754558 (NLRP3), miR-223 loss	ASC speck formation, Caspase-1 cleavage	Cytosol near mitochondria	Microglia, meningeal macrophages	NLRP3-GFP reporter mice, CX3CR1-CreERT2 lines	Low (post-priming)	MCC950, caspase-1 inhibitors	[67,68]
**REST/NRSF Complex**	Wnt, Notch, BDNF repression	Aging, inflammation, epigenetic drift	REST overexpression, CpG methylation, miR-124 loss	SUMOylation, MeCP2 binding	Perinucleolar chromatin domains	Hippocampal neurons, striatal interneurons	Aged mice, REST-GFP reporters, MeCP2 KO	High in early stages	REST siRNA, CoREST inhibitors	[69,70,71]
**Tau Kinase Hub**	GSK-3β, CDK5, MARK	Aβ exposure, insulin resistance	MAPT mutations, exon 10 splicing dysregulation	AT8 phospho-sites, acetylation K274	Axoplasm, dendritic spines	Cortical layer V neurons	P301S tau mice, TBI models, CSF tau proteomics	Low	Anti-tau ASOs, pan-kinase inhibitors	[72,73]
**MeCP2/MBD2**	Histone code–DNA methylation scaffold	ROS, metabolic instability	MECP2 duplication, Rett syndrome variants	HDAC3 tethering, phosphorylation-dependent dissociation	Nucleosome interface	Neurons, glial progenitors	MeCP2-null mice, iPSC-derived glia	Moderate	CRISPR-editing, MeCP2-stabilizing peptides	[74,75,76]
**PINK1–Parkin Gate**	Mitochondrial depolarization, calcium spikes	MPTP, ROS, dopaminergic stress	PARK2, PINK1 loss-of-function mutations	Ubiquitin phosphorylation, Mfn2 degradation	Outer mitochondrial membrane	SNpc dopaminergic neurons, astrocytes	Pink1/Parkin KO mice, iPSC-derived midbrain neurons	Moderate	Mitofusin agonists, autophagosome flux enhancers	[77,78]
**FUS/TDP-43 Nucleocytoplasmic Shuttling**	DNA damage, stress granule signaling	Oxidative DNA breaks, RNA instability	TARDBP, FUS mutations (ALS, FTD)	Phosphorylation, sumoylation, LLPS dynamics	Nucleoplasm ↔ cytosol, stress granules	Cortical and spinal motor neurons	ALS-FUS mice, iPSC neurons, C9ORF72-ALS lines	Low if LLPS already seeded	Phase separation inhibitors, nuclear transport correctors	[79,80]
**CDK5–p25 Hyperactivation Axis**	Ca^2+^ overload, NMDA excitotoxicity	ROS, Aβ, ischemia	p35 → p25 proteolytic shift, CDK5 mislocalization	Phosphorylation of tau, neurofilaments	Axon initial segment, perinuclear ER	Projection neurons, Purkinje cells	Ischemia models, AD postmortem cortex	Moderate	CDK5 inhibitors, calpain blockade	[81,82]
**Dicer/miRNA Processing Node**	miRNA biogenesis, synaptic plasticity	Inflammation, nuclear–cytoplasmic transport dysfunction	DICER1 loss in ALS, miRNA-132 repression	Phosphorylation, Dicer–TRBP interaction	Cytoplasmic P-bodies	Neurons, astrocytes, NSCs	Dicer KO models, Drosha-DGCR8 pathway studies	High (if early)	miRNA mimics, Dicer stabilization peptides	[83,84]
**Lamin B1–Nuclear Scaffold Axis**	Heterochromatin maintenance, nuclear integrity	Aging, oxidative damage, histone loss	LMNB1 overexpression in AD, epigenetic erosion	Phosphorylation, caspase cleavage	Nuclear lamina, chromatin contact zones	Neurons, OPCs, ependymal cells	Human AD tissue, lamin-deficient models	Low if fragmentation present	Lamin stabilizers, nuclear membrane chaperones	[85,86]

This table outlines the key signaling nodes where multiple upstream stressors converge and may influence the progression of neurodegenerative processes. It highlights context-dependent modulators, subcellular localization, and cell-type specificity, while also noting experimental models and potential avenues for targeted intervention. The intention is not to be exhaustive, but to provide a conceptual starting point for understanding how complex signaling collapse might be traced to distinct molecular junctions with varying degrees of reversibility. TSC1/2—Tuberous Sclerosis Complex 1 and 2; RHEB—Ras homolog enriched in brain; IRS1—Insulin receptor sub-strate 1; ULK1—Unc-51-like autophagy activating kinase 1; KO—Knockout; TFEB—Transcription factor EB; CTNNB1—Catenin beta 1 (β-catenin); MAPT—Microtubule-associated protein tau; APP/PS1—Amyloid precursor protein / Presenilin 1; CREBBP—CREB-binding protein; CaMKIIα—Calcium/calmodulin-dependent protein kinase II alpha; ROS—Reactive oxygen species; IL-1β—Interleukin-1 beta; 5hmC—5-hydroxymethylcytosine; miR-223—MicroRNA-223; ASC—Apoptosis-associated speck-like protein containing a CARD; CX3CR1—CX3C chemokine receptor 1; CreERT2—Cre recombinase-estrogen receptor T2 fusion; MCC950—NLRP3 inflammasome inhibitor; REST—RE1-silencing transcription factor; BDNF—Brain-derived neurotrophic factor; miR-124—MicroRNA-124; MeCP2—Methyl CpG binding protein 2; CoREST—Corepressor for REST; CDK5—Cy-clin-dependent kinase 5; MARK—Microtubule affinity-regulating kinase; AT8—Antibody recognizing phospho-tau (Ser202/Thr205); P301L—Pathogenic tau mutation; ASO—Antisense oligonucleotide; HDAC3—Histone deacetylase 3; MeCP2-null—MeCP2 knockout; PINK1—PTEN-induced kinase 1; Mfn2—Mitofusin 2; TARDBP—TAR DNA-binding protein 43; ALS—Amyotrophic lateral sclerosis; FTD—Frontotemporal dementia; C9ORF72—Chromosome 9 open reading frame 72; p25—Truncated CDK5 activator protein; ER—Endoplasmic reticulum; DICER1—Endoribonuclease Dicer; miRNA-132—MicroRNA-132; TRBP—TAR RNA-binding protein; DGCR8—DiGeorge syndrome critical region 8; LMNB1—Lamin B1.

**Table 2:** Representative regulatory axes that contribute to the molecular collapse observed in neurodegenerative contexts.

Regulatory Axis	Primary Failure Mode	Representative Disorders	Triggering Vulnerabilities	Biological Interface Affected	Omics-Derived Markers/Readouts	Regulatory Buffers/Adaptive Nodes	Precision Restoration Vectors	References
**Signal Integration Collapse**	Temporal desynchronization of intracellular pathways; feedback breakdown	AD, PD, ALS, HD	Oxidative stress, Aβ, cytokine storms	Neuron–glia axis	Phospho-proteomics; scRNA-seq signaling clusters	PTEN, IRS-1, phosphatases, CaMKII	CRISPR logic circuits; mTOR auto-tuners	[136,137,138]
**Epigenetic Drift and Noise Amplification**	Cell identity erosion; enhancer–promoter detachment	AD, ALS, FTD	Inflammation, chromatin erosion, tau	Transcriptional hubs	ATAC-seq entropy, MeCP2 loss, histone code shifts	DNMT1/3A, REST, SIRT1, CTCF	dCas9 editing, chromatin loop reweaving	[139,140]
**Immune Regulation Failure**	Chronic glial activation, failure of resolution pathways	AD, PD, ALS	Mito-DAMPs, APOE4, lipid imbalance	Microglia, astrocytes	scRNA-seq glial state trajectories; cytokine proteomics	TREM2, PPARγ, IL-10	TREM2 agonists, NLRP3 inhibitors, CD33 modulation	[141,142]
**Proteostasis–Glymphatic Collapse**	Clearance pathway overload; AQP4 polarity loss	AD, ALS, CAA	Tauopathy, ER stress, venous outflow failure	Interstitial matrix, astrocytic endfeet	Spatial proteomics; AQP4 mislocalization maps	TFEB, LAMP2A, HSPs, AQP4/α-syntrophin	AAV-TFEB, sleep-timed drainage therapies	[143,144,145]
**Degenerative Fate Lock-in**	Terminal glial or hybrid cell states; loss of neurogenic potential	ALS, PD, MS	NF-κB loops, Notch dysregulation, chromatin closure	Spatially restricted glial niches	Pseudotime bifurcation (scRNA-seq); trajectory fate traps	REST, Sox2, bHLH TFs, lamins	Astrocyte-to-neuron reprogramming; spatial CRISPR tools	[146,147,148]
**Synaptic Vesicle Regulation Failure**	Loss of vesicle cycling, endocytosis; neurotransmission collapse	ALS, PD, FTD	SNARE dysfunction, vesicle acidification, α-synuclein	Pre-/post-synaptic terminals	Synaptic proteome decay; VGLUT misexpression	Synaptotagmin, dynamin, Rab3A, Munc18	SV2A stabilizers, SNARE complex stabilizers	[149,150]
**Mitochondrial Network Collapse**	Fragmentation, fusion–fission imbalance, ROS overflow	ALS, PD, AD	PINK1/Parkin loss, calcium overload, mtDNA damage	Soma, axon terminals, astrocytes	Mito-tracker imaging; OXPHOS transcript depletion	MFN2, DRP1, SIRT3, OPA1	Mitofusin activators, NAD^+^ boosters, mitochondrial editing	[151,152]
**Neurovascular Uncoupling**	BBB breakdown, endothelial de-differentiation	AD, MS, stroke	Pericyte loss, chronic inflammation, hypoxia	Endothelium, perivascular glia	Spatial transcriptomics (CLDN5 loss); leakage assays	ZO-1, claudins, PDGFRβ, VEGF-A	AAV-mediated BBB repair; zonulin inhibitors	[153,154]
**Lipidomic Disintegration**	Lipid raft destabilization, myelin loss, peroxidation cascades	AD, MS, ALS	Cholesterol efflux imbalance, ferroptosis	Membranes, myelin, ER	Lipidomics; ferroptosis transcriptomics	ApoE, ABCA1, GPX4, PLA2	Ferroptosis inhibitors, lipidome modulators	[155,156]

Each axis reflects upstream stressors, genetic vulnerability, and subcellular localization, offering insights into the multidimensional nature of collapse and potential points of therapeutic engagement. AD—Alzheimer’s disease; PD—Parkinson’s disease; ALS—Amyotrophic lateral sclerosis; HD—Huntington’s disease; CAA—Cerebral amyloid angiopathy; MS—Multiple sclerosis; Mito—Mitochondria; DAMPs—Damage-associated molecular patterns; scRNA-seq—Single-cell RNA sequencing; ATAC-seq—Assay for Transposase-Accessible Chromatin with high-throughput sequencing; MeCP2—Methyl CpG binding protein 2; DNMT1/3A—DNA methyltransferases 1 and 3A; REST—RE1-silencing transcription factor; SIRT1—Sirtuin 1; dCas9—Catalytically dead Cas9; PPARγ—Peroxisome proliferator-activated receptor gamma; IL-10—Interleukin-10; NLRP3—NOD-, LRR- and pyrin domain-containing protein 3; TFEB—Transcription factor EB; LAMP2A—Lysosome-associated membrane glycoprotein 2A; AAV-TFEB—Adeno-associated virus–mediated TFEB; HSPs—Heat shock proteins; AQP4—Aquaporin 4; Sox2—SRY-Box Transcription Factor 2; NF-κB—Nuclear factor kappa-light-chain-enhancer of activated B cells; SNARE—Soluble NSF Attachment Protein Receptor; VGLUT—Vesicular glutamate transporter; SV2A—Synaptic vesicle glycoprotein 2A; Rab3A—Ras-related protein Rab-3A; Munc18—Mammalian uncoordinated-18 (syntaxin-binding protein 1); mtDNA—Mitochondrial DNA; OXPHOS—Oxidative phosphorylation; MFN2—Mitofusin 2; DRP1—Dynamin-related protein 1; SIRT3—Sirtuin 3; OPA1—Optic atrophy 1; BBB—Blood–brain barrier; ZO-1—Zonula occludens-1; Claudins—Tight junction proteins Claudins; AAV—Adeno-associated virus; PDGFRβ—Platelet-derived growth factor receptor beta; VEGF—Vascular endothelial growth factor; ABCA1—ATP-binding cassette transporter A1; GPX4—Glutathione peroxidase 4; PLA2—Phospholipase A2.

## Data Availability

The data presented in this study are available on request from the corresponding author.
